# A Novel Peptide Antibiotic Produced by *Streptomyces roseoflavus* Strain INA-Ac-5812 With Directed Activity Against Gram-Positive Bacteria

**DOI:** 10.3389/fmicb.2020.556063

**Published:** 2020-09-15

**Authors:** Alexey S. Vasilchenko, William T. Julian, Olda A. Lapchinskaya, Genrikh S. Katrukha, Vera S. Sadykova, Eugene A. Rogozhin

**Affiliations:** ^1^Laboratory of Antimicrobial Resistance, Institute of Environmental and Agricultural Biology (X-BIO), Tyumen State University, Tyumen, Russia; ^2^Gause Institute of New Antibiotics, Moscow, Russia; ^3^Shemyakin and Ovchinnikov Institute of Bioorganic Chemistry, Russian Academy of Sciences, Moscow, Russia

**Keywords:** peptide antibiotic, lipoglycopeptide antibiotic, *Streptomyces roseoflavus*, daptomycin, anti-biofilm action, calcium-depending antibiotic

## Abstract

In this work, we report the isolation and detailed functional characterization for the new non-ribosomally synthesized antibiotic 5812-A/C, which was derived from metabolites of *Streptomyces roseoflavus* INA-Ac-5812. According to its chemical structure, the studied 5812-A/C preliminary is composed of a cyclic peptide part covalently bounded with an arabinose residue. N-terminal amino acid sequencing of the native peptide has identified its partial structure of Leu-Asp-Gly-Ser-Gly and consisting of a Tyr residue that is supposed to have a two-component peptide nature for the molecule studied. However, the structural analysis of the antibiotic complex derived from *S. roseoflavus* INA-Ac-5812 is still ongoing. The mechanism of action of 5812-A/C was assessed in comparison with its most related analog, the lipopeptide antibiotic daptomycin, given the presence in both antimicrobials of an L-kynurenine amino acid residue. The inhibitory activity of 5812-A/C against Gram-positive bacteria including methicillin-resistant strain of *Staphylococcus aureus* was similar to daptomycin. The mechanism of action of 5812-A/C was associated with the disruption of membrane integrity, which differs in comparison with daptomycin and is most similar to the antimicrobial membrane-disturbing peptides. However, 5812-A/C demonstrated a calcium-dependent mode of action. In addition, unlike daptomycin, 5812-A/C was able to penetrate mature biofilms and inhibit the metabolic activity of embedded *S. aureus* cells. At the same time, 5812-A/C has no hemolytic activity toward erythrocyte, but possessed weak cytotoxic activity represented by heterochromatin condensation in human buccal epithelium cells. The biological properties of the peptide 5812-A/C suggest its classification as a calcium-dependent antibiotic effective against a wide spectrum of Gram-positive pathogenic bacteria.

## Introduction

The discovery and study of new antibiotics can help avert the threat of antimicrobial resistance as well as provide new information about the microworld and its properties. Peptide antibiotics are one of the most interesting groups of antimicrobial substances for many reasons, especially their rapid onset of antimicrobial activity that prevents a chance for the selection of resistant bacterial cells. Among peptide antibiotics, there is a group of nonribosomal peptides (NRPs) (Schwarzer et al., 2003). NRPs are characterized as having non-proteinogenic amino acids or a non-peptide moiety linked with a peptide component. In 2019, the existing database of NRPs contained more than 1,730 NRPs (Caboche et al., 2008). The structural and compositional variety of these peptides allows them to have a broad range of important biological activities.

NRP antibiotics can be classified as glycopeptides, lipopeptides, or glycolipopeptides depending on the nature of the non-peptide component. One of the features of some lipopeptide antibiotics is a strict dependence of antimicrobial activity on the presence of calcium ions in the medium. This has led to the creation of a separate class of calcium-dependent lipopeptides (CDAs), with over 40 unique CDAs currently identified (Wood and Martin, 2019). Daptomycin is the first cyclic lipopeptide approved in the United States by the Food and Drug Administration (FDA). Daptomycin is a calcium-dependent antibiotic, which have been taken to clinical trials, and has different mechanisms of action (Vilhena and Bettencourt, 2012). Several models of daptomycin’s antimicrobial action have been proposed, including membrane pore formation (Beriashvili et al., 2018), alteration of membrane fluidity (Muller et al., 2016), and inhibition of cell wall biosynthesis (Müller et al., 2016). It is indeed possible that all proposed models are representative of the action of daptomycin. Other known lipopeptides, such as amphomycin, friulimicin, and malacidin, have mechanisms of action involving the inhibition of bacterial cell wall biosynthesis through binding to lipid II (malacidins) (Hover et al., 2018) or bactoprenol phosphate carrier C_55_-P (friulimicin) (Schneider et al., 2009).

Glycopeptide and glycolipopeptide antibiotics exert an antimicrobial effect by interfering with cell wall biosynthesis. This phenomenon may include inhibiting the trans-glycosylation step of peptidoglycan biosynthesis (ramoplanin; Lo et al., 2000) or interactions with the terminal *D*-alanyl-*D*-alanine moieties of the NAM/NAG-peptides (vancomycin; Reynolds and Somner, 1990).

The novel antibiotic compound studied was firstly purified from a culture medium of actinomycete *Streptomyces* sp. INA-5812 (Gause Institute of New Antibiotics collection) and partially characterized and designated as 5812-A/C (5812-1; Lapchinskaya et al., 2016). The substance is presumably a cyclic (lipo)glycopeptide, with a molecular weight of 1,916.8 Da. The antibiotic contains an arabinose residue, nine amino acids (one residue of Asp/Asn, Ser, Pro, Ala, Leu, Tyr, and Orn, two residues of Gly), and three non-identified amino acid residues. Acidic hydrolysis of the molecule, followed by fluorescence excitation/emission values and high resolution mass spectrometry of INA-5812, revealed the presence of 4-chloro-L-kynurenine (Alferova et al., 2018).

Interestingly, the first evaluation of the 5812-A/C antimicrobial spectra revealed the inhibition of the growth of some Gram-negative bacteria (*Prtoteus vulgaris* 206) (Lapchinskaya et al., 2016).

The aim of this work is to provide a detailed evaluation of the biological activity of 5812-A/C including determining its bactericidal spectra and assessing the antibiotic’s mode of action using *Staphylococcus aureus* 209P as a model microorganism.

## Materials and Methods

### Strains, Media, and Antibiotics

*Streptomyces roseoflavus* INA-Ac-5812 from the Collection of Microorganisms is producer of the studying antibiotic 5812-A/C (Gause Institute of New Antibiotics, Moscow, Russia). Cultivation was carried out according to the described method (Timofeeva et al., 2012).

Tested bacterial strains included *Staphylococcus aureus* (MRSA: oxacillin-, methicillin-resistant), *Enterococcus faecium* ICIS 18, *E. faecium* 79OSAU, *Staphylococcus aureus* 209P (MSSA: methicillin-susceptible), *Bacillus cereus* IP 5832, *Escherichia coli* K12, *E. coli* MG1655, *Pseudomonas aeruginosa* ATCC 28753, *Salmonella enterica* ATCC 14028, *Chromobacterium violaceum* ATCC 31532, and *Pectobacterium carotovorum* VKM-B1247. Bacteria were grown to mid-log phase in cation-adjusted Mueller-Hinton broth (Becton Dickinson, United States) supplemented with CaCl_2_ for 0.45 mM. Daptomycin, vancomycin, and nisin were obtained from Sigma-Aldrich (United States).

### Isolation of 5812-A/C From the Culture Medium

The antibiotic complex INA-5812 was extracted from culture media after *S. roseoflavus* cultivation (centrifuged at 7,000 rpm, 10 min, 4°C) by first separation on a XAD-2 resin (Serva, United Kingdom) equilibrated with ultrapure water, followed by the extraction of the unbound liquid by n-butanol (1:1, w/w). The obtained extract was evaporated, dissolved in 60% EtOH, and applied to an XBridge C18 BEH column (4.6 × 250 mm, 130 Å, 5 μm) for further separation by analytical reversed-phase high-performance liquid chromatography (HPLC). Fractionation was carried out at a linear gradient of acetonitrile concentration from 8 to 40% with 0.1% trifluoroacetic acid (TFA) for 50 min at a flow rate of 950 μl/min. Absorbance was detected at 214 and 364 nm. The target fraction was collected manually. Re-chromatography of the target peak was carried out using analytical reversed-phase HPLC on a Synergi Polar-RP 100 Å 4 μm 3 × 100 mm (Phenomenex, United States) column in a linear gradient of acetonitrile:2-propanol (80:20, w/w) concentration from 10 to 50% for 15 min at a flow rate of 0.42 ml/min. Detection was monitored at 214 nm (Supplementary Figure 1). Re-chromatography found the purity of the 5812-A/C preparation to be 93.2%.

### Spectrophotometric Analysis

UV/Visible spectrum of purified 5812-A/C preparation was measured on a Shimadzu UV-1800 (Shimadzu Corp., Japan) in a scan range of 200–800 nm.

### MALDI TOF/TOF Mass Spectrometry

The molecular mass of the peptide was measured by a matrix-assisted laser desorption/ionization (MALDI) time-of-flight (TOF) mass spectrometry on an Autospeed MALDI-TOF instrument (Bruker Daltonics, Germany) in positive ion mode. 2,5-Dihydroxybenzoic acid (Sigma-Aldrich, United States) was used as a matrix. Mass spectra were analyzed using the FlexAnalysis software (Bruker Daltonics, Germany).

### Edman Sequencing

Automated N-terminal sequencing was performed on a PSSQ-33A sequencer (Shimadzu Corp., Japan) according to the manufacturer’s protocol. Identification of amino acid residues was conducted as PTH derivatives using the LabSolutions software.

### Determination of Antibacterial Activity

Determination of minimal inhibitory concentration (MIC) was performed as described in methods (Fuchs et al., 2000; Mascio et al., 2007), following the Clinical and Laboratory Standards Institute guidelines for broth microdilution MIC assays. Cation-adjusted Mueller-Hinton II broth (Becton Dickinson, Sparks, MD, United States) was supplemented with an additional CaCl_2_ for 0.45 mM (CAMHBc). Bacteria were incubated in a 96-well microtiter plate (Eppendorf, Germany) containing 90 μl of inoculum prepared in growth media at ∼10^6^ CFU/ml with 10 μl of twofold dilutions of the antibiotics. The dynamics of bacterial growth were assessed by scanning and plotting the absorbance data at 620 nm obtained by spectrophotometer (Multiscan GO, Thermo Fisher Scientific, United States). The cell contents with no visible bacterial growth were transferred to LB agar by the drop plate approach (Sharafutdinov et al., 2017). The antimicrobial activity of the antibiotic was measured with respect to both the MIC and the minimal bactericidal concentration (MBC). MIC was determined as the lowest concentration of antibiotic for which no visible bacterial growth could be observed after 24 h of incubation, whereas MBC was defined as the lowest concentration of antimicrobial that will prevent the growth of an organism after culturing on antibiotic-free media.

The dependence of the antimicrobial activity of antibiotics on the content of calcium cations in the medium was evaluated using LB broth (ApplyChem, Germany) supplemented with CaCl_2_ at a concentration range of 0–0.9 mM.

### Permeabilization of the Plasma Membrane and Microscopy

Membrane integrity was assessed using the fluorescent probes SYTO 9 and propidium iodide (PI) (LIVE/DEAD BacLight Bacterial Viability Kit, Molecular Probes, United States). The test strain *S. aureus* 209P was grown to the mid-log phase in CAMHBc. The obtained culture was centrifuged at 10,000 rpm for 10 min, the supernatant was discarded, and the pellet was dispersed in 10 mM HEPES buffer supplemented with 0.5% glucose and 0.45 mM CaCl_2_ to an optical density of 0.2 (OD_620_) that corresponded to 10^8^ CFU/ml. Bacterial cells in 50 μl were transferred to a 96-well microtiter plate (Eppendorf, Germany). Measurements of SYTO 9 fluorescence kinetics were performed on a Fluoroskan Ascent FL plate reader (Thermo Fisher Scientific, United States) at 485 nm excitation and 535 nm emission wavelengths as described earlier (Rogozhin et al., 2018). Assessment of the viability of bacterial cells was performed by the agar-drop plate assay using one part of the treated bacterial population. Another part of the treated bacteria was used to prepare samples for fluorescence microscopy. Fluorescence microscopy was performed using a Zeiss Axio Imager A2 fluorescent microscope (Carl Zeiss, Germany) equipped with filter sets useful for simultaneous viewing of SYTO 9 and PI fluorescence.

### Antibiofilm Activity of 5812-A/C and Daptomycin

#### Preforming and Treatment of *S. aureus* Biofilms

Biofilms of *S. aureus* 209P were grown as described in reference (Frapwell et al., 2020) with some modifications. The cultures of *S. aureus* 209P were grown in flat-bottomed 96-well microtiter plates (Eppendorf, Germany) in LB broth supplemented with glucose (0.5%) and yeast extract (5 g/L). The microplates were incubated at 37°C for 20 h, followed by the discarding of the medium and addition of the fresh medium. Following 60 h of incubation, planktonic cells were removed from each well, and the plates were rinsed twice using sterile HEPES buffer (10 mM, 0.45 mM CaCl_2_). One hundred microliters of each antibiotic dissolved in HEPES buffer was added into the wells in twofold serial dilutions. Microtiter plates were incubated at 25°C for 3 h. After treatment, the antibiotic solutions were discarded, and the wells were washed two times with HEPES buffer.

#### Evaluation of the Metabolic Activity of Biofilm-Embedded Bacteria Using the Tetrazolium/Formazan Assay

The assay was performed as described previously with some modifications (Sabaeifard et al., 2014). Solutions of 2,3,5-triphenyl-tetrazolium chloride (TTC) (DiaM, Russia) were prepared by dissolving TTC in distilled water in concentrations of 1% and then sterilized by filtration through 0.22 μm PVDF filters (Millipore, United States). Fifty microliters of TTC solution and 200 μl of CAMHBc were added to the treated and untreated biofilms formed in the wells of microtiter plates. Plates were incubated in the dark for 5 h at 37°C. After incubation, the well contents were removed, and 95% ethanol was added for 10 min. Dissolved formazan was removed to a new flat-bottomed microplate, and the absorbance was measured at 490 nm.

#### Evaluation of Biofilm Eradication Using Crystal Violet Binding Assay

The biomass of the grown biofilms was quantified using a crystal violet staining procedure (O’Toole, 2011). Hundred microliter of aqueous crystal violet solution (0.1%) was added to each well and incubated at room temperature (22–24°C). The staining solution was then removed, and the wells were washed twice with deionized water to remove excess dye. Dye that was strongly bound to the biofilms was solubilized with 100 μl of 95% ethanol for 10 min. Quantitative analysis of crystal violet absorbance was measured using paired samples and analyzed at 540 nm using a plate reader.

### Hemolytic Activity

Hemolytic assay was performed as described previously (Oddo and Hansen, 2017)^.^ Freshly drawn human erythrocytes were rinsed three times with 0.9% solution of NaCl and re-suspended in the same buffer to 1% (v/v). One hundred microliters of the suspension was added to a 96-well microtiter plate containing equal volume of antibiotic to give final concentrations of peptides encompassing the range of 50–0.1 μg/ml (A_A_). Buffer and double distilled water were used as negative control (A_NC_) and positive control (A_PC_, 100% hemolysis), respectively. Indolicidin has been introduced as an additional positive control. Plates were incubated at 37°C for 1 h. Subsequently, the plate was centrifuged, and the supernatant was transferred to a new plate. The release of hemoglobin in the supernatant was monitored at an absorbance of 414 nm using a Multiscan GO plate reader (Thermo Fisher Scientific, United States). The resulting values of optical absorption were processed according to Equation (1):

(1) $$\mathrm{Hemolysis}:\left(\left({\text{A}}_{\text{A}}^{414}-{\text{A}}_{\text{NC}}^{414}\right)/\left({\text{A}}_{\text{PC}}^{414}-{\text{A}}_{\text{NC}}^{414}\right)\right)\times 100\%$$

### Influence on Heterochromatin Condensation in Human Buccal Epithelium Cells

Quantitative analysis of heterochromatin granules located in the nuclei of human buccal epithelium cells was performed as described previously (Shckorbatov et al., 1998). 5812-A/C was applied at concentrations ranging from 1.25 to 10.0 μg/ml.

Prepared samples of antibiotics were dissolved in pure sterile water (18 MΩ) and stored at −20°C prior to their use in antimicrobial assays.

### Statistical Analysis

The experiments were performed using two independent series with three technical replicates each. The obtained results were statistically analyzed using the Origin 2015 software (OriginLab Corporation, Northampton, MA, United States). The difference significances were estimated with the paired sample Wilcoxon signed rank test. The differences were significant at *p* < 0.05.

## Results

### Isolation of 5812-A/C and Partial Structure Analysis

**FIGURE 1:**
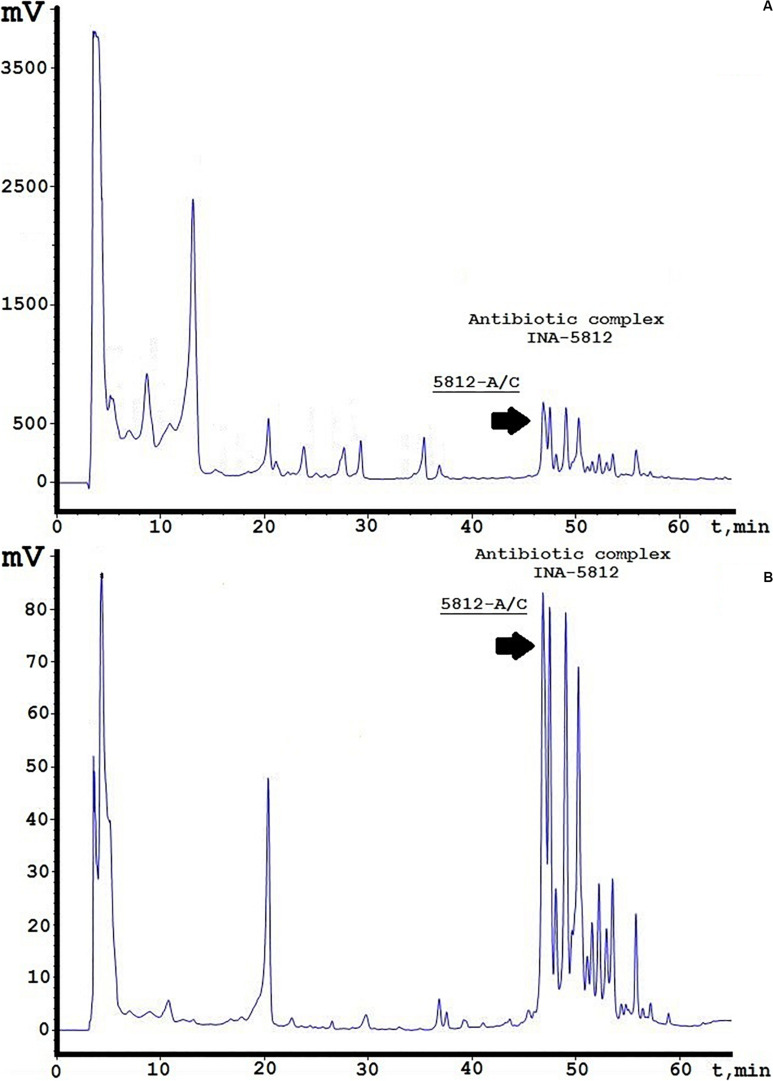
Analytical reversed-phase HPLC analysis of INA-5812 antibiotic concentrate. **(A)** A profile is presented at 214 nm; **(B)** at 364 nm. Elution peak corresponding to 5812-A/C is marked by a black arrow.

A concentrated antibiotic solution was obtained from culture liquid using a two-stage purification process. One liter of centrifuged medium was first applied to a XAD-2 column, and the unbound fraction was collected for further separation. The second stage consisted of liquid-phase extraction with n-butanol using a separating funnel. The obtained butanolic extract was fully evaporated, resolved in 60% ethanol solution, and analyzed by reversed-phase HPLC. Figure 1 illustrates the complete spectrum of compounds isolated from the extract by their detection at two UV wavelengths (214/364 nm). It should be noted that detection at 214 nm is typical for peptide bonds, whereas 364 nm was selected based on the UV absorbance spectrum of 5812-A/C as recorded earlier (Alferova et al., 2018). The relative diversity of the obtained compounds suggests that a simplified method of extraction would be acceptable for obtaining the INA-5812 fraction, as no admixtures were found to disturb the elution of the target peptide (Figure 1A). Detection at 364 nm could also discover a set of components containing preliminary the same chromophore and located in the diapason from 45 to 60 min (Figure 1B), and there are more than 16 peaks detected, including five major peaks. 5812-A/C (or 5812-1; Alferova et al., 2018), which was selected for detailed structural–functional analysis, is the first compound from this group eluted from the column and is dominant on adsorption level. The molecular mass was confirmed by MALDI TOF/TOF MS and has an average value of 1,916.8 Da (data not presented). Structural analysis gave the purity of the fraction acquired by analytical reversed-phase HPLC as greater than 92%. Five hundred picomolar of 5812-A/C was analyzed by automated Edman degradation, and 11 cycles were sequenced. As a result, we were able to estimate a short amino acid sequence of Leu-Asp-Gly-Ser-Gly and the presence of a Tyr residue that may be associated with the other peptide part of the molecule (Supplementary Figure 1); thus, a cyclic architecture could be possible for the 5812-A/C substance. Deeper investigation of its 2D structure with NMR imaging is planned for the future.

### Antimicrobial Activity of 5812-A/C Against Gram-Positive and Gram-Negative Bacteria

The antibiotic 5812-A/C has an antimicrobial effect against Gram-positive microorganisms. In most cases, the MIC was found to be the same as the MBC (Table 1). The bactericidal activity of 5812-A/C toward *S. aureus* 209P and *B. cereus* IP5812 was comparable to the lipopeptide daptomycin and the glycopeptide vancomycin, which are expected to be structural analogs of the studied substance. The susceptibility of *S. aureus* MRSA and MSSA strains to 5812-A/C is fivefold different. However, 5812-A/C was not effective against *E. faecium* strains applied in the entire range of concentrations taken (Table 1). 5812-A/C was also ineffective against all tested Gram-negative strains, which were resistant to all tested concentrations (Table 1).

**TABLE 1:** Spectra of 5812-A/C activity comprising known peptide antibiotics.

Bacterial cell wall	Organisms	Drugs	Minimal inhibitory concentrations, μg/ml	Minimal bactericidal concentrations, μg/ml
Gram-positive	*Staphylococcus aureus* 209P (MSSA)	5812-A/C	0.8	0.8
		Daptomycin	0.8	0.8
		Vancomycin	3.8	3.8
		Nisin	7.5	7.5
	*Staphylococcus aureus* (MRSA)	5812-A/C	3.1	3.1
		Daptomycin	0.7	0.7
		Vancomycin	3.7	3.7
		Nisin	1.9	1.9
	*Enterococcus faecium* ICIS 18	5812-A/C	>120	>120
		Daptomycin	8.1	8.1
		Vancomycin	1.6	1.6
		Nisin	7.5	7.5
	*Enterococcus faecium* 79OSAU	5812-A/C	>120	>120
		Daptomycin	12.5	12.5
		Vancomycin	0.7	0.7
		Nisin	7.5	7.5
	*Bacillus cereus* IP 5832	5812-A/C	3.8	7.5
		Daptomycin	4.1	8.1
		Vancomycin	0.8	0.8
		Nisin	3.9	7.8
Gram-negative	*Escherichia coli* K12	5812-A/C	60	>120
		Daptomycin	>120	>120
		Vancomycin	>100	>100
		Nisin	62.5	>125
	*Escherichia coli* MG1655	5812-A/C	>120	>120
		Daptomycin	>120	>120
		Vancomycin	>100	>100
		Nisin	>125	>125
	*Pseudomonas aeruginosa* ATCC 28753	5812-A/C	>120	>120
		Daptomycin	>120	>120
		Vancomycin	>100	>100
		Nisin	>125	>125
	*Salmonella enterica* ATCC 14028	5812-A/C	>120	>120
		Daptomycin	>120	>120
		Vancomycin	>100	>100
		Nisin	>125	>125
	*Chromobacterium violaceum* ATCC 31532	5812-A/C	>120	>120
		Daptomycin	>120	>120
		Vancomycin	>100	>100
		Nisin	>125	>125
	*Pectobacterium carotovorum* VKM-B1247	5812-A/C	>120	>120
		Daptomycin	>120	>120
		Vancomycin	>100	>100
		Nisin	>125	>125

### Effect of Calcium on 5812-A/C Activity

**FIGURE 2:**
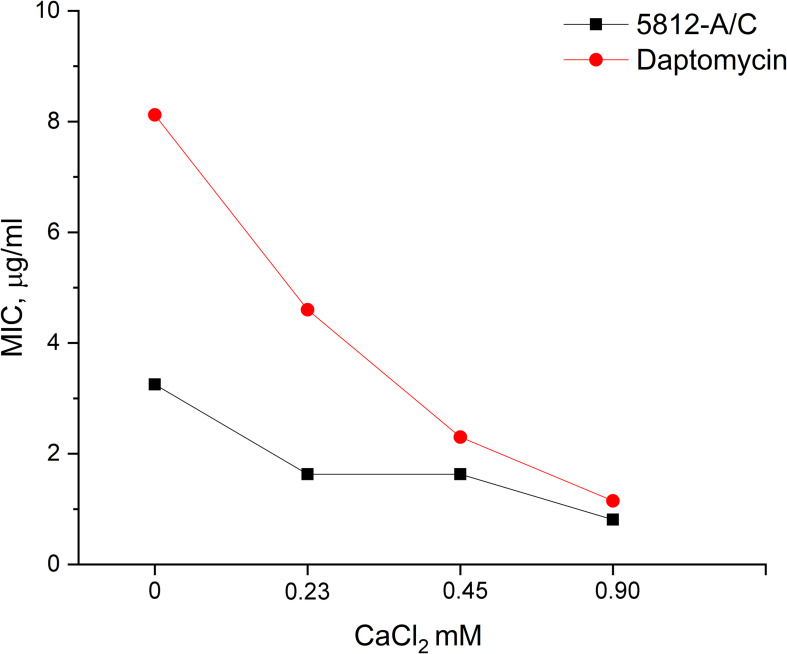
The dependence of the inhibitory activity of antibiotics on the concentration of calcium in the medium. The MIC of 5812-A/C and daptomycin against *S. aureus* 209P was assessed at various concentrations of calcium, and the inhibition of 5812-A/C was found to be calcium-dependent.

Functional similarities between the structure of 5812-A/C and daptomycin dictated the need to evaluate the possible dose-dependent effect of calcium cations on the value of MICs. We found that MIC values for daptomycin decreased eightfold (from 8.12 to 1.02 μg/ml) as calcium cation content concentration increased from 0 to 0.9 mM (Figure 2). In turn, the biological activity of 5812-A/C also turned out to be calcium dependent. The introduction of CaCl_2_ up to 0.9 mM reduces the MIC value by four times (from 3.25 to 0.81 μg/ml) (Figure 2).

#### Deciphering the Mode of Action of 5812-A/C

Application of the fluorescent probes SYTO 9 and PI allowed for the evaluation of 5812-A/C’s effect on membrane permeability. The sequential introduction of fluorophores and antibiotics into the reaction medium made it possible to assess the dynamic changes in the integrity of the bacterial cytoplasmic membrane in real time.

**FIGURE 3:**
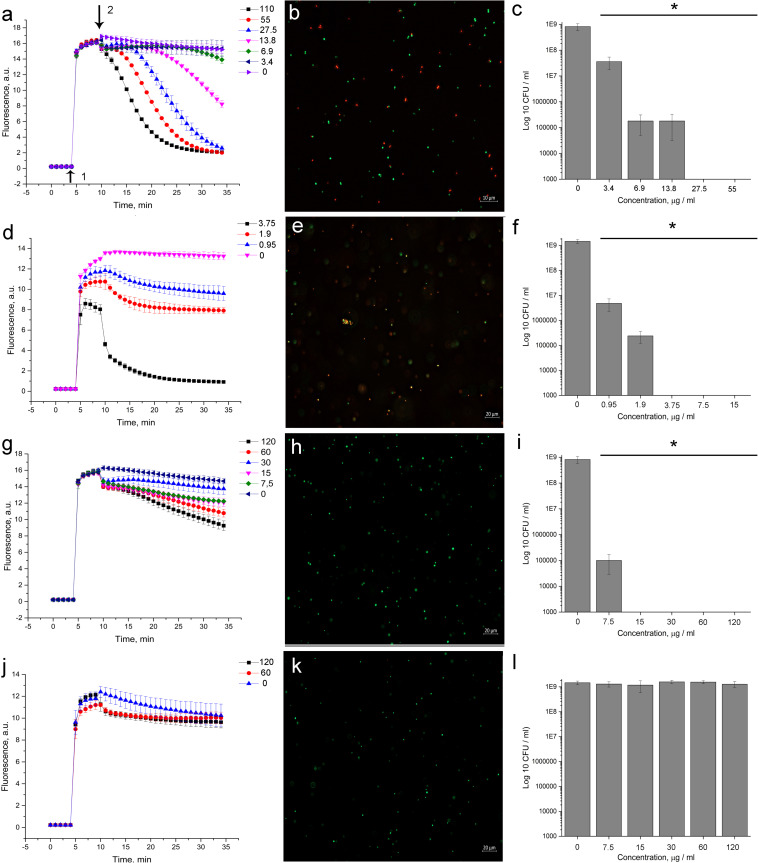
Evaluation of membrane disturbance of *S. aureus* 209P using Live/Dead fluorescent dyes and agar-drop assay. **(a,d,g,j)** Intensity of SYTO 9 fluorescence. If bacterial membranes are permeabilized, propidium iodide penetrates into the cell. What follows is SYTO 9 getting displaced from nucleic acids, which leads to a decrease in fluorescence intensity in the green region (505–540 nm) of the spectrum. **(b,e,h,k)** Epifluorescence microscopy images of *S. aureus* 209P after 1 h of treatment. **(c,f,i,l)** Cell’s viability after exposure with antibiotics for 1 h. 5812-A/C **(a–c)**, nisin **(d–f)**, daptomycin **(g–i)**, vancomycin **(j–l)**. 1—The moment of introduction of the fluorophore; 2—the moment of introduction of the antibiotic. *Indicate significant differences at *p* < 0.05.

The introduction of 5812-A/C led to a quenching of SYTO 9 fluorescence (Figure 3a) in a dose-dependent manner. Complete inhibition of SYTO 9 fluorescence occurred within 30 min. A similar effect was recorded with the addition of nisin (Figure 3d), which has well-known membrane permeabilizing properties. Daptomycin affected the kinetics of SYTO 9 fluorescence in a dose-dependent manner, but complete quenching was not achieved (Figure 3g). Vancomycin did not show any activity in this assay (Figure 3j).

Following the measurement of SYTO 9 fluorescent kinetics, the viability of the bacteria was evaluated by agar-drop plating. It was found that a 1-hour exposure of bacterial cells to 5812-A/C, daptomycin, and nisin (Figures 3c,f,i, respectively) was sufficient to kill all bacteria in the reaction mixture, but vancomycin did not demonstrate bactericidal activity (Figure 3l).

Notably, the observed effect of 5812-A/C (Figure 3a) and nisin (Figure 3d) on membrane integrity was consistent with a reduction of bacterial viability at the same concentrations (Figures 3c,f, respectively, for both antibiotics). Exposure of bacteria to daptomycin for MBC (15 μg/ml) (Figure 3i) was accompanied by slightly pronounced quenching of SYTO 9 fluorescence (Figure 3g). Vancomycin did not inhibit SYTO 9 fluorescence nor cell viability during hourly exposure (Figures 3j,i) at any concentrations used.

The permeabilizing effect of 5812-A/C has been clearly shown through fluorescence microscopy. Treated *S. aureus* populations become heterogeneous, consisting of both intact cells (green object) as well as cells with damaged membranes (red objects) (Figure 3b). Similar results were recorded with nisin (Figure 3e), but not daptomycin and vancomycin (Figures 3h,k, respectively).

### Bactericidal Effect of 5812-A/C Against Preformed Biofilm of *S. aureus* 209P

The inhibitory effect of 5812-A/C was evaluated not only against planktonic cells but also against bacteria embedded in stress-tolerant bacterial biofilms.

**FIGURE 4:**
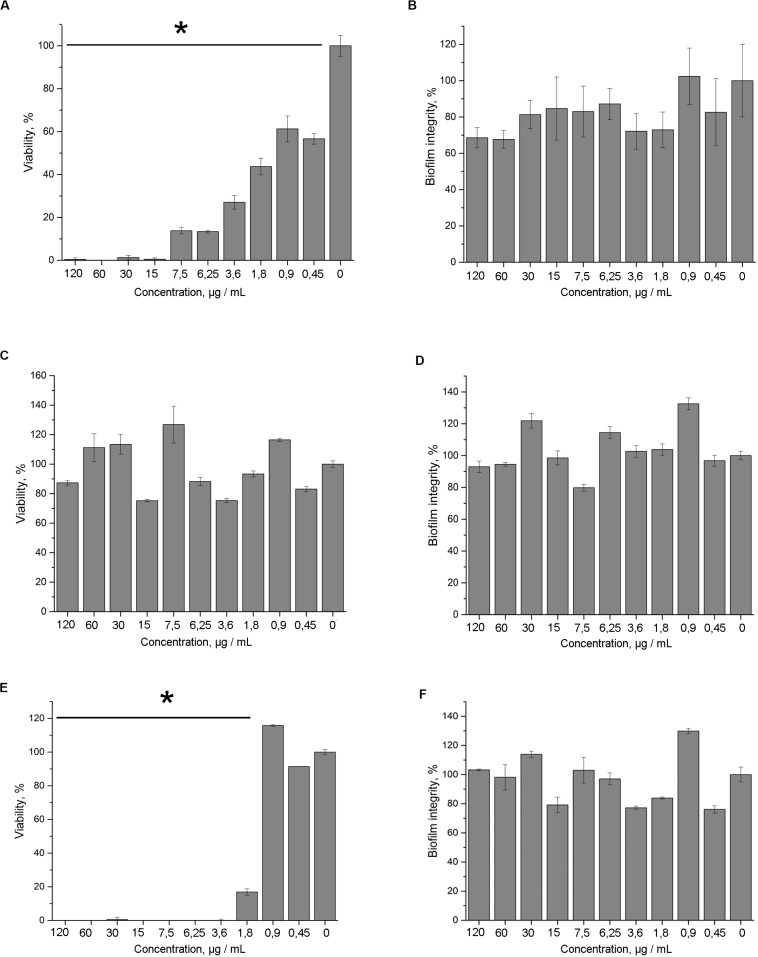
Antimicrobial activity of 5812-A/C against *S. aureus* 209P in preformed biofilms. Inhibition of the metabolic activity (TTC assay) of *S. aureus* cells, which are embedded in biofilm matrix compared with untreated samples. Treatment with: 5812-A/C **(A)**, daptomycin **(C)**, nisin **(E)**; biofilm biomass (crystal violet assay) after the treatment with: 5812-A/C **(B)**, daptomycin **(D)**, nisin **(F)**. *Indicate significant differences at *p* < 0.05.

5812-A/C was found to effectively inhibit the bacterial metabolic activity in mature biofilms compared with intact samples. 5812-A/C was sufficient to inhibit the enzymatic activity of biofilm-embedded cells at a concentration of 15 μg/ml (Figure 4A). This is similar to the action of nisin (Figure 4E). The lack of daptomycin effectiveness against bacterial biofilms was somewhat unexpected (Figure 4C). Daptomycin concentrations that were sufficient to kill planktonic bacterial cells within an hour were found to be ineffective against biofilm-embedded bacteria. In turn, none of the antibiotics taken in the experiment demonstrated the ability to eradicate mature biofilms. The results of the crystal violet assay showed a lack of destructive effect on the biofilm for 5812-A/C and other antibiotics (Figures 4B,D,F).

### *In vitro* Hemolytic Properties of 5812-A/C

**FIGURE 5:**
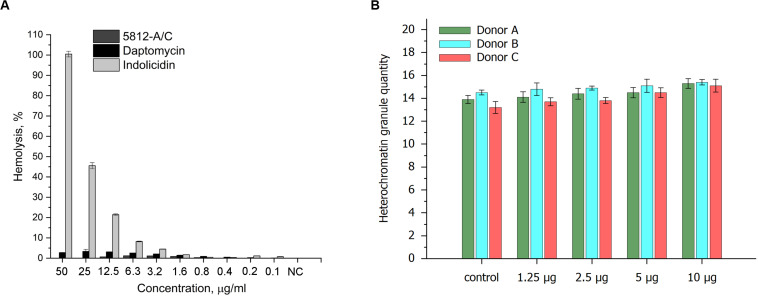
Cytotoxic properties of 5812-A/C in relation to prokaryotic cells. Hemolytic activity of 5812-A/C, daptomycin, and indolicidin to human erythrocyte **(A)**. Designations: NC—negative control (0.9% solution of NaCl); assay was repeated in triplicate, and percentage of hemolysis is expressed as mean ± SD. Influence of 5812-A/C on heterochromatin condensation located in the nuclei of human buccal epithelium cells **(B)**.

Taking into account the membranolytic activity of 5812-A/C against bacterial cells, we evaluated the ability of 5812-A/C to interact with the membrane of mammalian cells. The hemolytic effects of 5812-A/C were compared with daptomycin and indolicidin as an additional positive control. During an hour of incubation with 5812-A/C and daptomycin, no significant lysis of erythrocytes was recorded. Thus, incubation of erythrocytes with 5812-A/C for 50 μg/ml led to lysis of less than 1% of erythrocytes. In turn, daptomycin lysed around 3% of erythrocytes at the same concentration, whereas antimicrobial cationic peptide indolicidin showed the dose-dependent ability to lyse of 100–5% of erythrocytes in a concentration range of 50–3.2 μg/ml (Figure 5A).

### Influence of 5812-A/C on Heterochromatin Condensation in Human Buccal Epithelium Cells

We also investigated the possible influence of the peptide antibiotic on heterochromatin condensation located in the nuclei of human buccal epithelium cells. The data obtained partially correspond to previous results and show weak heterochromatin granule formation at 10 μg compared with control values (Figure 5B). Consequently, the cytotoxicity shown could be associated with the interaction of 5812-A/C with DNA, despite the fact that it is inactive against fungi and yeast, anionic molecules, but is able to internalize into a cell. These results are consistent with previously reported data where 5812-A/C was completely inactive against eukaryotic microorganisms (*Aspergillus niger* and *Candida albicans*).

## Discussion

Actinomycetes are traditionally one of the most important sources for the discovery of new antibiotics (Genilloud, 2017). Screening to identify novel antimicrobial compounds with peptide moieties is quite significant, as few peptide antibiotics are known, and many of them are of interest in battling microbes resistant to conventional antibiotics (Gurnev and Nestorovich, 2014; Ling et al., 2015; Hong et al., 2019). *S. roseoflavus* is a well-known species that is attractive for researchers as a good source of novel antibiotics that can inhibit opportunistic pathogenic microorganisms (Park et al., 2006; Shi et al., 2018). *S. roseoflavus* INA strains are being widely studied to investigate a number of biologically active components with antimicrobial functions including the macrolide-type antibiotic irumamycin, which is active against opportunistic yeasts and fungi (Shashkov et al., 2011; Lapchinskaya et al., 2015). Previously, two structural homologs (isoirumamycin and the compound X-1495B) identified from INA-Ac-5812 strains and structurally characterized by NMR spectroscopy were found to have previously unknown stereo-configuration (Alferova et al., 2019).

In this study, we performed a structural and functional analysis of the antimicrobial peptide complex 5812-A/C produced by *S. roseoflavus* INA-Ac-5812 (Lapchinskaya et al., 2016). The peptide compound had strong bactericidal effect against Gram-positive bacteria. Initially, a procedure was developed to purify the antibiotic from the growth media by separation on an Amberlite XAD-2 resin, followed by the extraction of the unbound fraction with n-butanol. This approach minimized quantitative losses of the substance. The structural properties of the molecule were also investigated, and the exact molar mass, relative amino acid composition, and UV and fluorescence spectra were determined. Regarding structural elicitation, this compound was studied to determine an exact molecular mass, relative amino acid composition, UV and fluorescence spectra, and chemical derivatization (Lapchinskaya et al., 2016), which is earlier used for modification of some aminoglycoside antibiotics (Topolyan et al., 2016). At present, automated Edman degradation allowed for the determination of a partial structure of 5812-A/C, including a five amino acid fragment. The overall structure of the antibiotic is believed to be cyclic, and we speculate that the fragment results following the cleavage of some peptide bonds by waterless TFA as the second stage of N-terminal sequencing. We propose that two peptide bonds were cleaved, followed by the appearance of PTH-Leu and PTH-Tyr derivatives that were visualized. It can be explained by two free NH2 groups at once for further modification with phenylisothiocyanate (PITC) (Heinrikson, 1978). This conclusion is quite important for potential application of Edman degradation method for determination of non-linear peptide antibiotics produced by bacteria and actinomycetes.

The structure of 5812-A/C suggests that the antibiotic belongs to the group of glycopeptide compounds characterized by the incorporation of non-standard amino acid residues into the peptide chain. In addition to the peptide fragment, arabinose carbohydrate forms a major component structure of the molecule (Lapchinskaya et al., 2016). Due to these structural similarities, the new antibiotic INA 5812-A/C is believed to be an analog of vancomycin and daptomycin. However, preliminary investigation of antimicrobial spectra showed the activity of 5812-A/C against Gram-negative *P. vulgaris* (Lapchinskaya et al., 2016), which differed from both vancomycin and daptomycin. In this regard, it is obvious that the mechanism of action of the new antibiotic is also presumably different from its analogs.

The antimicrobial effect of 5812-A/C against Gram-positive microorganisms including a methicillin-resistant *S. aureus* strain was found to be comparable with antibiotics of “last hope” vancomycin and daptomycin. However, unlike these comparable antibiotics, 5812-A/C was ineffective against *E. faecium* strains. It is known that enterococci are generally less susceptible to lipopeptides than staphylococci (Miller et al., 2014). Differences in the composition of the cytoplasmic membrane of these two species likely determine sensitivity to 5812-A/C. This is corroborated by the results pertaining to the permeability of membranes, where enterococci were not compromised after exposure to the antibiotic.

We were not able to record the antimicrobial activity of 5812-A/C against several Gram-negative bacterial species tested. Thus, the antibiotic 5812-A/C is believed to not inhibit the viability of Gram-negative bacteria.

The mechanism of action of INA 5812-A/C showed some similarities with daptomycin. It shows a pronounced dependence of its bactericidal activity on the presence of calcium ions in the medium. Drawing parallels with daptomycin, it can be assumed that 5812-A/C molecule has a negative charge in aqueous solutions, as the initial isolation from raw growth media used an anion-exchange sorbent DEAE-Sephadex (Lapchinskaya et al., 2016). Interactions with Ca^2+^ ions are involved in neutralizing the anionic charges of the peptide (Rotondi and Gierasch, 2005). Binding of calcium is almost exclusively coordinated in proteins by oxygen ligands that originate in the side-chain carboxyl groups of Asp and Glu (Kirberger and Yang, 2013). Calcium-dependent antibiotics usually contain conserved Asp_5_-X_6_-Asp_7_-Gly_8_ calcium-binding motifs (Strieker and Marahiel, 2009; Wood et al., 2019). As was established by the Edman amino acid sequencing, 5812-A/C has a similar motif in the sequence Leu-Asp-Gly-Ser-Gly. Position 6 plays a key role in the calcium-binding motif (Asp_5_-X_6_-Asp_7_-Gly_8_), and its substitution plays a significant role in the antibacterial activity of the antibiotic molecule (Strieker and Marahiel, 2009). Daptomycin contains alanine at position 6 (Wood et al., 2019), whereas 5812-A/C contains leucine as was determined preliminarily. This might explain a certain difference in calcium-dependent bactericidal activity between daptomycin and 5812-A/C. Thus, on this basis, we believe that the new antibiotic 5812-A/C can be attributed to this small family of calcium-dependent antibiotics.

The disruption of membrane integrity was assessed using two fluorescent dyes, one of which penetrates the concentration gradient freely, and the second only in the presence of damage to the cytoplasmic membranes. It turned out that 5812-A/C exhibits the properties of a typical membrane-destroying antibiotic, which is reminiscent of the action of nisin (Vasilchenko and Valyshev, 2019). The main evidence is suggested by the quenching of SYTO 9 fluorescence due to the penetration of PI.

The antimicrobial effect of daptomycin acts through its interaction with the cytoplasmic membrane. However, this requires hours (Beriashvili et al., 2018), but not seconds like 5812-A/C. Recorded dissimilarity in the kinetic of permeabilization is a key difference between 5812-A/C and daptomycin in the mode of action.

The antibiotic 5812-A/C is able to effectively destroy planktonic cells of Gram-positive microorganisms, including methicillin-resistant *S. aureus*. However, many bacterial populations exist predominantly in the form of biofilms. It is known that in this state, many bacterial species become insensitive to the effects of antibiotics (Donlan, 2000).

Many antibiotics can prevent the formation of biofilms during initial cell adhesion, as cellular processes are quite sensitive to external factors at this stage (Cédric and Caillon, 2014). However, a mature biofilm is a tough nut for many antibiotics. Daptomycin has the ability to prevent the formation of staphylococcus biofilms only at the stage of cell surface adhesion (Mishra et al., 2015), but is ineffective against formed biofilms (Boudjemaa et al., 2016). In this regard, it is advisable to either use a combination of lipopeptide antibiotics with other agents (Parra-Ruiz et al., 2010) or use other peptide antibiotics that are capable of action on a mature biofilm (Mishra et al., 2015). 5812-A/C was able to penetrate and inhibit the metabolic activity of cells in mature biofilms at concentrations slightly higher than the concentration effective against planktonic cells. As a result, we can conclude that the new antibiotic is highly effective against all forms of existence of the bacterial population of *S. aureus*.

## Conclusion

A novel lipopeptide compound was isolated from 5812-A/C *Streptomyces* sp. INA-5812 and found to have structural and functional characteristics similar to other commercial lipopeptide antibiotics, including its presumed structural analog daptomycin. Some principal functional differences were found in comparison with daptomycin, including a direct effect on the cellular membrane, followed by rapid permeabilization and strong bactericidal effect on target populations. Moreover, 5812-A/C was uniquely able to inhibit the metabolism of bacterial cells associated with mature biofilms, which is interesting with respect to multidrug-resistant strains and clinical isolates.

## Data Availability Statement

The raw data supporting the conclusions of this article will be made available by the authors, without undue reservation.

## Author Contributions

AV and ER conceived, designed, and supervised the study. AV, WJ, and ER performed the experiments, analyzed and interpreted the data, and drafted and wrote the manuscript. OL, GK, and VS corrected the manuscript. All authors approved the final version of the manuscript.

## Conflict of Interest

The authors declare that the research was conducted in the absence of any commercial or financial relationships that could be construed as a potential conflict of interest.

## Supplementary Material

FIGURE S1 Purification of 5812-A/C: **(A)** re-chromatography of the peak obtained after separation of the total concentrate; **(B)** UV spectra of the purified antibiotic.
