# Supplementary figures and images for: A Novel Peptide Antibiotic Produced by Streptomyces roseoflavus Strain INA-Ac-5812 With Directed Activity Against Gram-Positive Bacteria
Source: Front Microbiol. 2020 Sep 15;11:556063. doi: 10.3389/fmicb.2020.556063 (PMC7533577; doi:10.3389/fmicb.2020.556063)

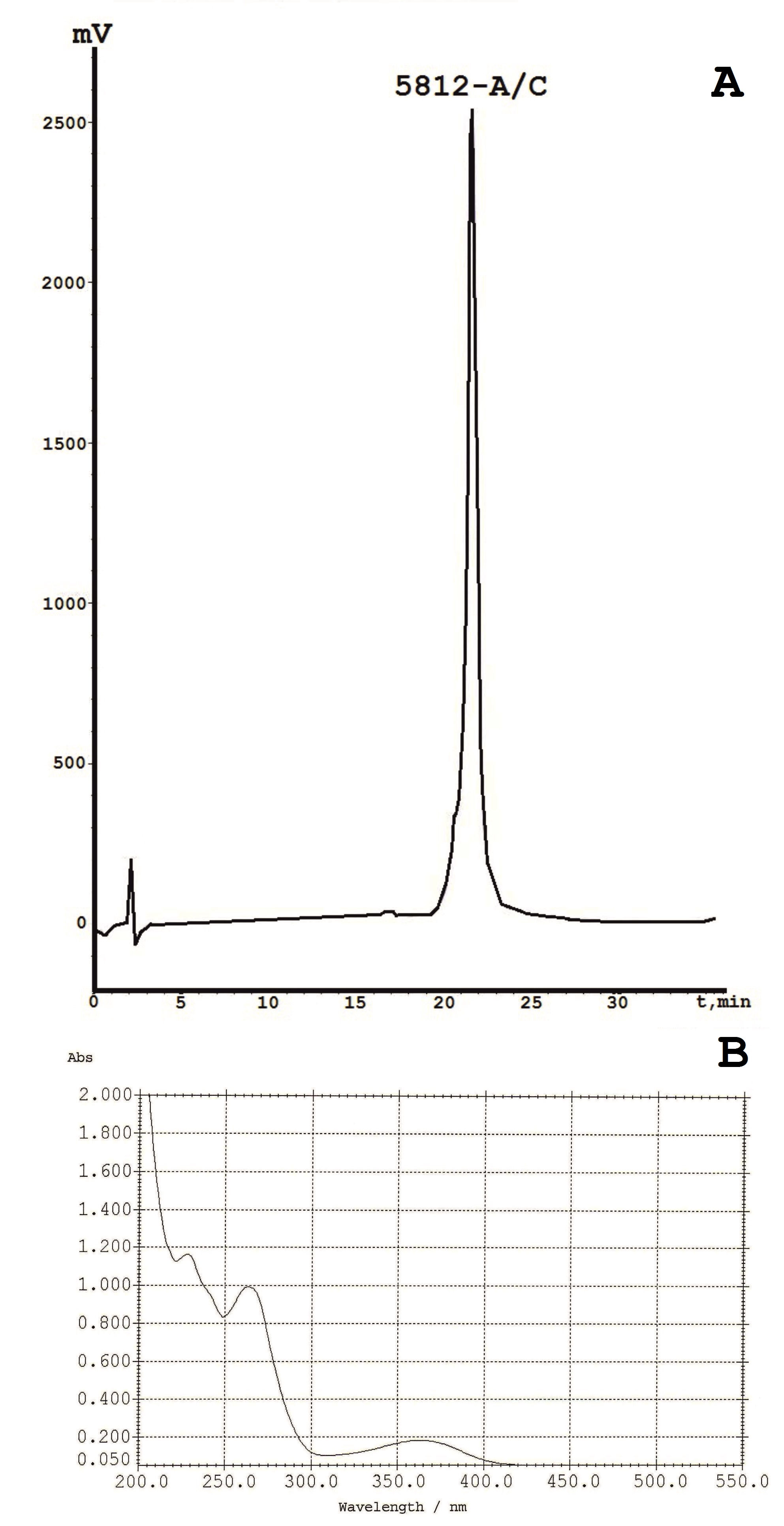

Supplement: FIGURE S1 — Purification of 5812-A/C: (A) re-chromatography of the peak obtained after separation of the total concentrate; (B) UV spectra of the purified antibiotic. [file Image_1.JPEG]
