# Supplementary material for: A Novel Peptide Antibiotic Produced by Streptomyces roseoflavus Strain INA-Ac-5812 With Directed Activity Against Gram-Positive Bacteria
Source: Front Microbiol. 2020 Sep 15;11:556063. doi: 10.3389/fmicb.2020.556063 (PMC7533577; doi:10.3389/fmicb.2020.556063)
Supplement: Supplementary file 2 [file Data_Sheet_1.PDF]

Data Filename : 5812A_070515+_02_D01.lcd
Sample Name : 5812-B
Method Filename : 5812A_070515+_02_SQ.LCM
Date Acquired : 07.05.2015 14:50:50
Background Filename :

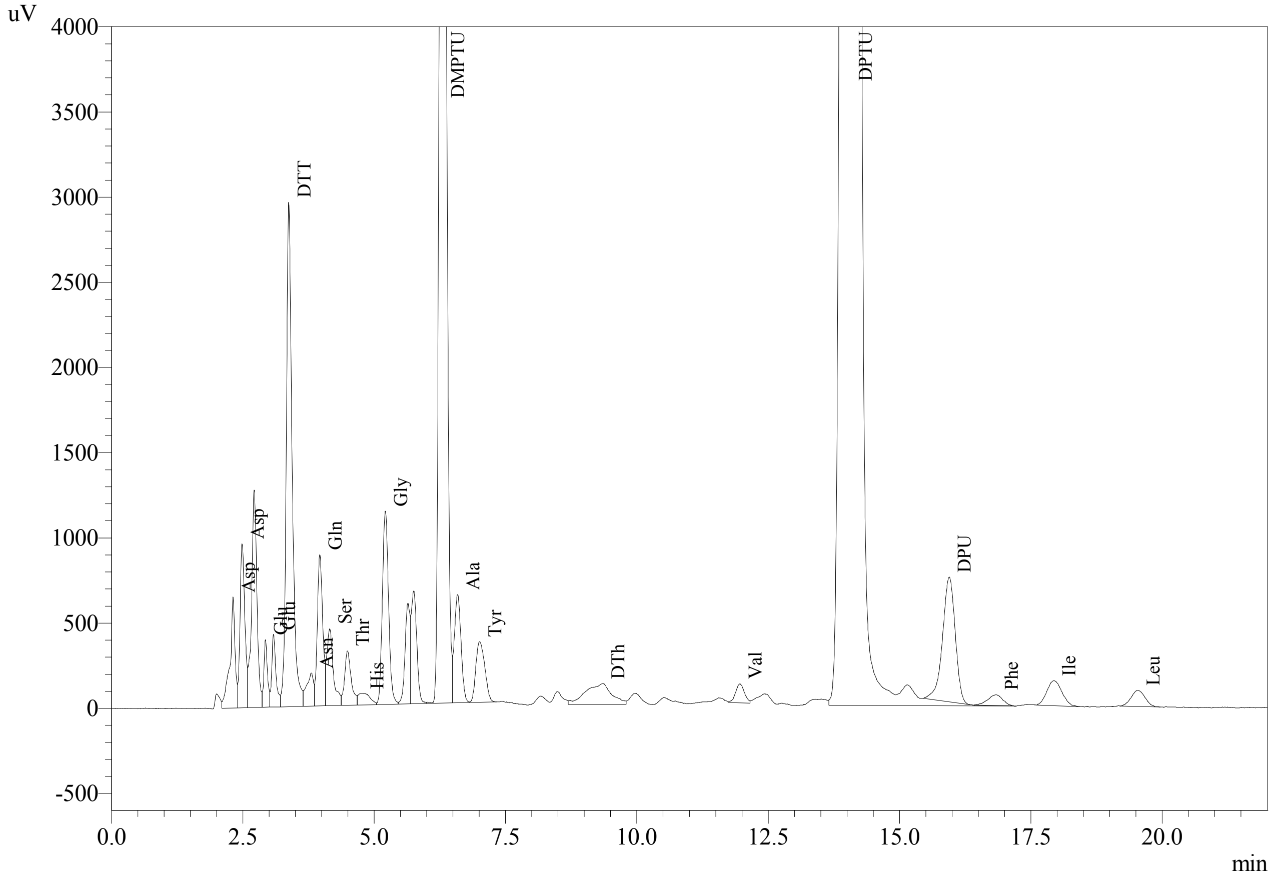

PeakTable
Detector A Ch1 269nm

Peak#	Name	Ret. Time	Area	Conc.
1	Asp	2.311	4535	8.823
2	Asp	2.484	6241	12.141
3		2.715	9086	
4	Glu	2.929	1916	4.194
5	Glu	3.082	2408	5.270
6	DTT	3.373	23164	
7	Asn	3.801	1857	4.055
8	Gln	3.963	6400	13.516
9	Ser	4.151	3920	12.274
10	Thr	4.490	2819	7.332
11	His	4.762	1080	2.402
12	Gly	5.211	9326	24.328
13		5.640	4038	
14		5.751	4738	
15	DMPTU	6.299	86490	
16	Ala	6.585	5526	13.234
17	Tyr	7.005	4218	10.059
18	DTh	9.348	4284	27.322
19	Val	11.957	1295	2.872
20	DPTU	14.055	894865	
21	DPU	15.942	12327	
22	Phe	16.837	1199	2.946
23	Ile	17.935	2907	6.325
24	Leu	19.533	1722	3.815
Total			1096358	

Data Filename : 5812A_070515+_02_D02.lcd
Sample Name : 5812-B
Method Filename : 5812A_070515+_02_SQ.LCM
Date Acquired : 07.05.2015 15:39:13
Background Filename :

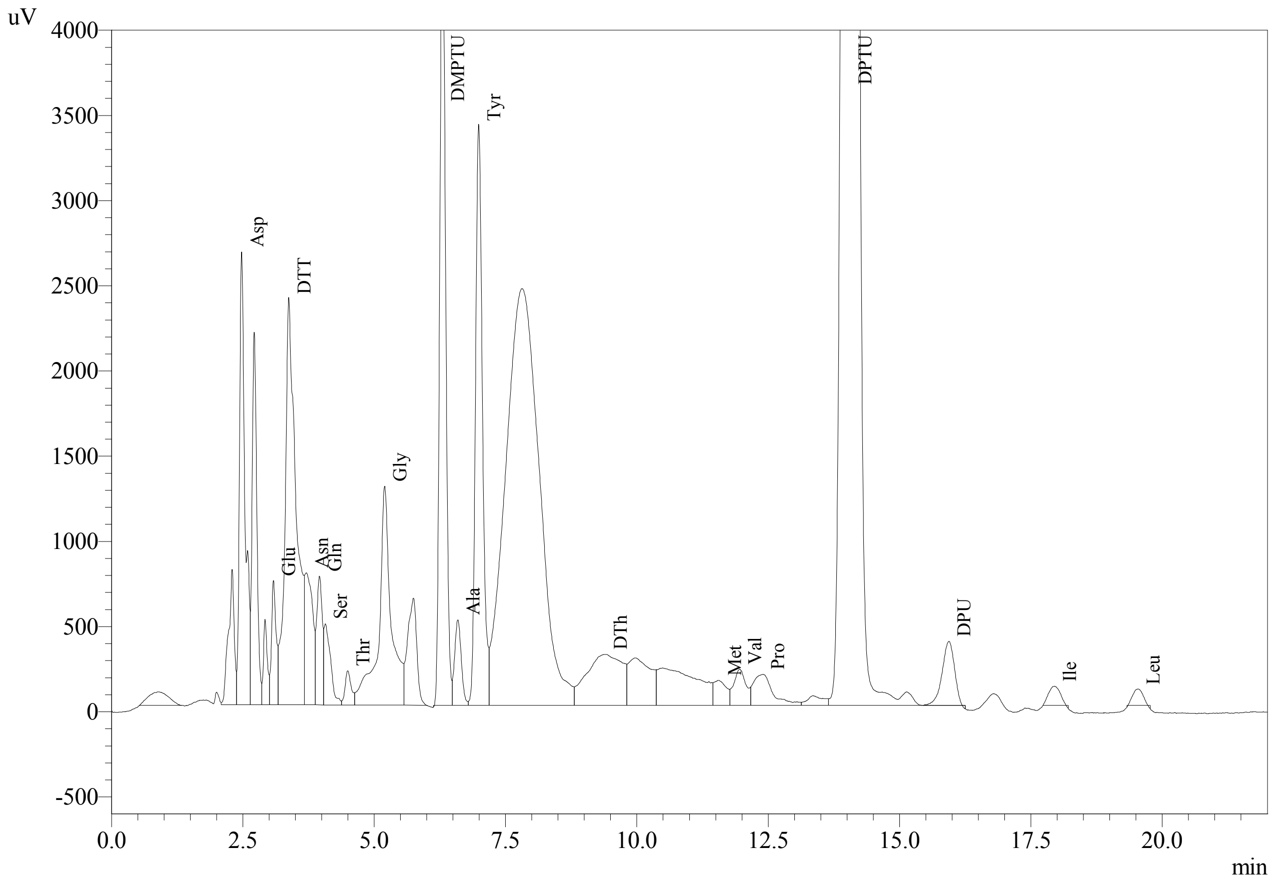

PeakTable
Detector A Ch1 269nm

Peak#	Name	Ret. Time	Area	Conc.
1		0.902	2128	
2		2.294	6129	
3	Asp	2.475	19944	38.800
4		2.715	13718	
5		2.921	2663	
6	Glu	3.080	4496	9.841
7	DTT	3.372	35188	
8	Asn	3.710	8270	18.060
9	Gln	3.957	5705	12.050
10	Ser	4.069	4127	12.923
11	Thr	4.494	1671	4.347
12	Gly	5.198	21397	55.819
13		5.744	7585	
14	DMPTU	6.299	36829	
15	Ala	6.590	4390	10.514
16	Tyr	6.987	31581	75.311
17		7.813	109432	
18	DTh	9.397	14095	89.891
19		9.969	8163	
20		10.530	11507	
21	Met	11.570	2405	5.331
22	Val	11.955	3496	7.754
23	Pro	12.389	4635	12.842
24		13.354	1218	
25	DPTU	14.051	477410	
26	DPU	15.935	6096	
27	Ile	17.943	1718	3.738
28	Leu	19.533	1283	2.843

Peak#	Name	Ret. Time	Area	Conc.
Total			847280	

Data Filename	: 5812A_070515+_02_D03.lcd
Sample Name	: 5812-B
Method Filename	: 5812A_070515+_02_SQ.LCM
Date Acquired	: 07.05.2015 16:27:36
Background Filename	:

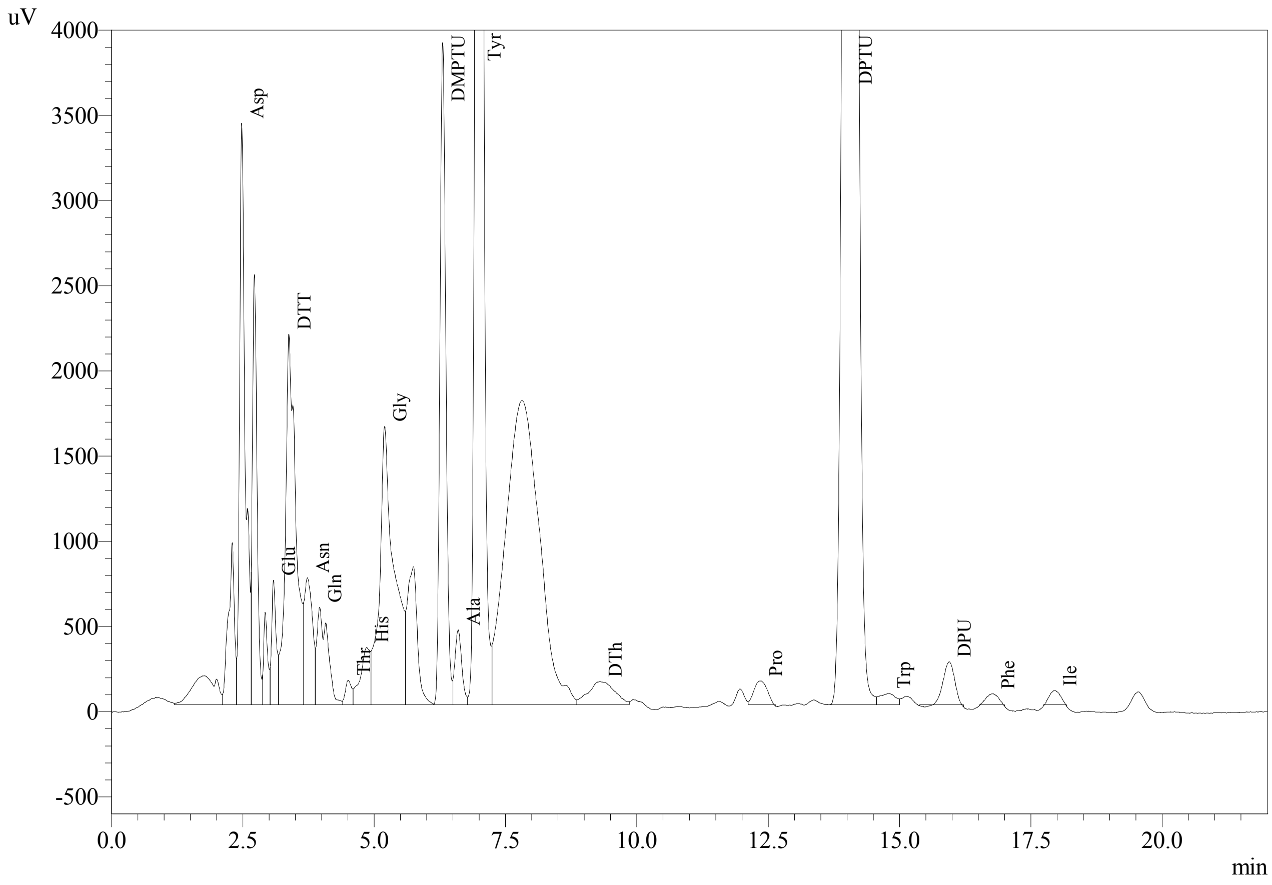

PeakTable
Detector A Ch1 269nm

Peak#	Name	Ret. Time	Area	Conc.
1		1.765	5532	
2		2.295	7649	
3	Asp	2.478	26442	51.443
4		2.719	15386	
5		2.926	2914	
6	Glu	3.083	4488	9.823
7	DTT	3.375	30511	
8	Asn	3.729	7867	17.181
9	Gln	3.960	8083	17.072
10	Thr	4.504	1198	3.116
11	His	4.855	4448	9.900
12	Gly	5.198	31844	83.071
13		5.742	10659	
14	DMPTU	6.303	30673	
15	Ala	6.596	3985	9.545
16	Tyr	6.991	115144	274.581
17		7.812	79645	
18	DTh	9.294	4741	30.234
19	Pro	12.350	2450	6.789
20	DPTU	14.056	296939	
21	Trp	14.788	1402	2.941
22	DPU	15.942	3839	
23	Phe	16.769	1026	2.521
24	Ile	17.951	1208	2.627
Total			698073	

Data Filename	: 5812A_070515+_02_D04.lcd
Sample Name	: 5812-B
Method Filename	: 5812A_070515+_02_SQ.LCM
Date Acquired	: 07.05.2015 17:15:59
Background Filename	:

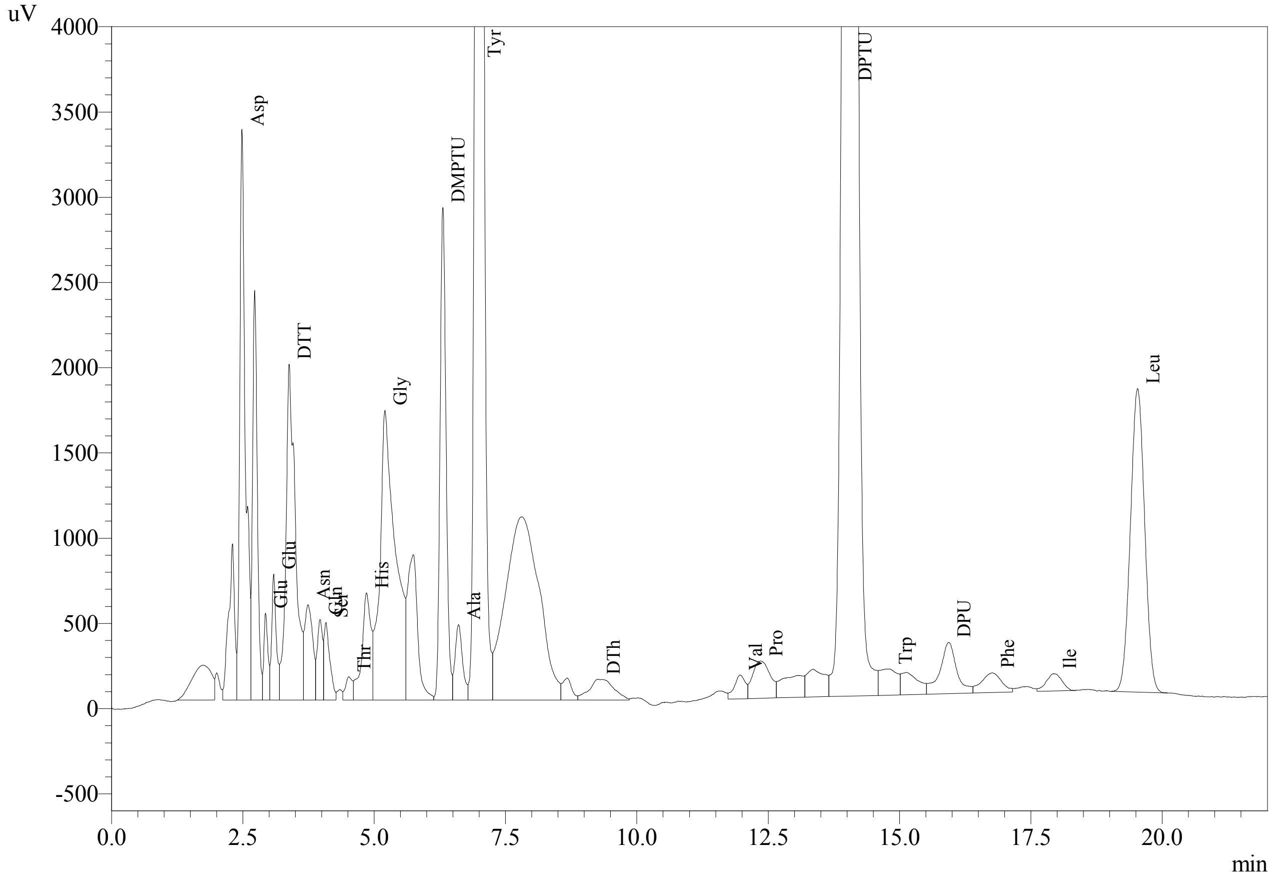

PeakTable
Detector A Ch1 269nm

Peak#	Name	Ret. Time	Area	Conc.
1		1.740	5351	
2		2.300	7431	
3	Asp	2.481	25478	49.566
4		2.724	15019	
5	Glu	2.930	2784	6.092
6	Glu	3.085	4525	9.903
7	DTT	3.380	24729	
8	Asn	3.738	5994	13.089
9	Gln	3.968	3403	7.188
10	Ser	4.079	3497	10.950
11	Thr	4.515	1253	3.259
12	His	4.851	7584	16.878
13	Gly	5.203	35045	91.422
14		5.742	11398	
15	DMPTU	6.305	23161	
16	Ala	6.603	4426	10.600
17	Tyr	6.991	137588	328.102
18		7.808	48218	
19		8.670	1577	
20	DTh	9.257	3912	24.949
21	Val	11.963	2087	4.630
22	Pro	12.357	5151	14.273
23		13.035	3813	
24		13.349	3920	
25	DPTU	14.050	268408	
26	Trp	14.815	3605	7.562
27		15.155	2984	
28	DPU	15.933	6747	

Peak#	Name	Ret. Time	Area	Conc.
29	Phe	16.759	3116	7.656
30	Ile	17.943	2129	4.633
31	Leu	19.528	33657	74.581
Total			707990	

Data Filename : 5812A_070515+_02_D05.lcd
Sample Name : 5812-B
Method Filename : 5812A_070515+_02_SQ.LCM
Date Acquired : 07.05.2015 18:04:24
Background Filename :

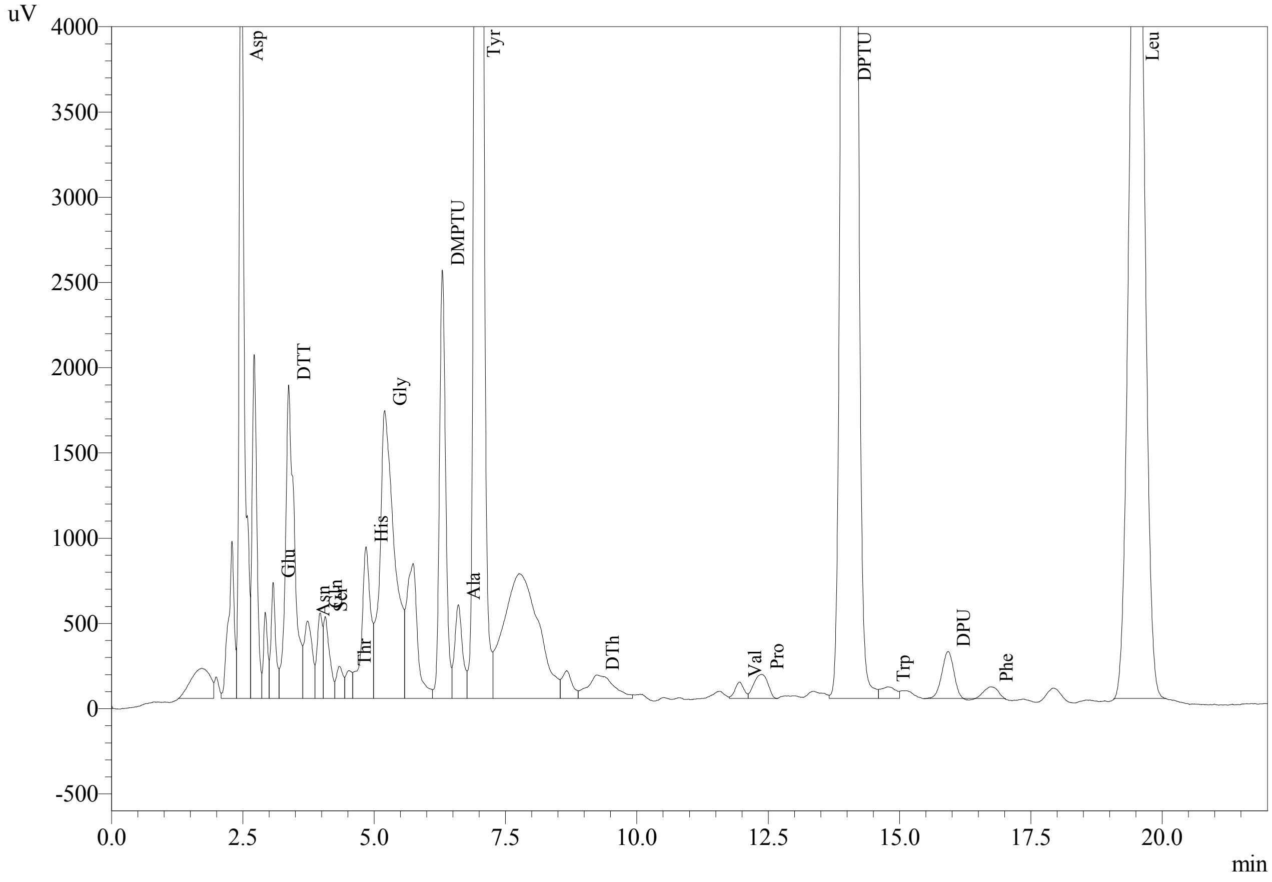

PeakTable
Detector A Ch1 269nm

Peak#	Name	Ret. Time	Area	Conc.
1		1.723	4608	
2		2.292	7200	
3	Asp	2.465	33900	65.952
4		2.715	12697	
5		2.925	2820	
6	Glu	3.074	4354	9.529
7	DTT	3.370	21600	
8	Asn	3.730	4871	10.637
9	Gln	3.969	3734	7.887
10	Ser	4.066	3845	12.040
11		4.335	1732	
12	Thr	4.519	1374	3.573
13	His	4.842	10810	24.058
14	Gly	5.198	33211	86.638
15		5.737	11407	
16	DMPTU	6.296	20816	
17	Ala	6.599	5826	13.954
18	Tyr	6.982	141026	336.302
19		7.759	34168	
20		8.661	2220	
21	DTh	9.241	4790	30.551
22	Val	11.953	1143	2.536
23	Pro	12.374	2609	7.230
24	DPTU	14.038	298181	
25	Trp	14.774	1377	2.888
26	DPU	15.921	4385	
27	Phe	16.737	1285	3.158
28	Leu	19.514	113843	252.266

Peak#	Name	Ret. Time	Area	Conc.
Total			789833	

Data Filename : 5812A_070515+_02_D06.lcd
Sample Name : 5812-B
Method Filename : 5812A_070515+_02_SQ.LCM
Date Acquired : 07.05.2015 18:52:47
Background Filename :

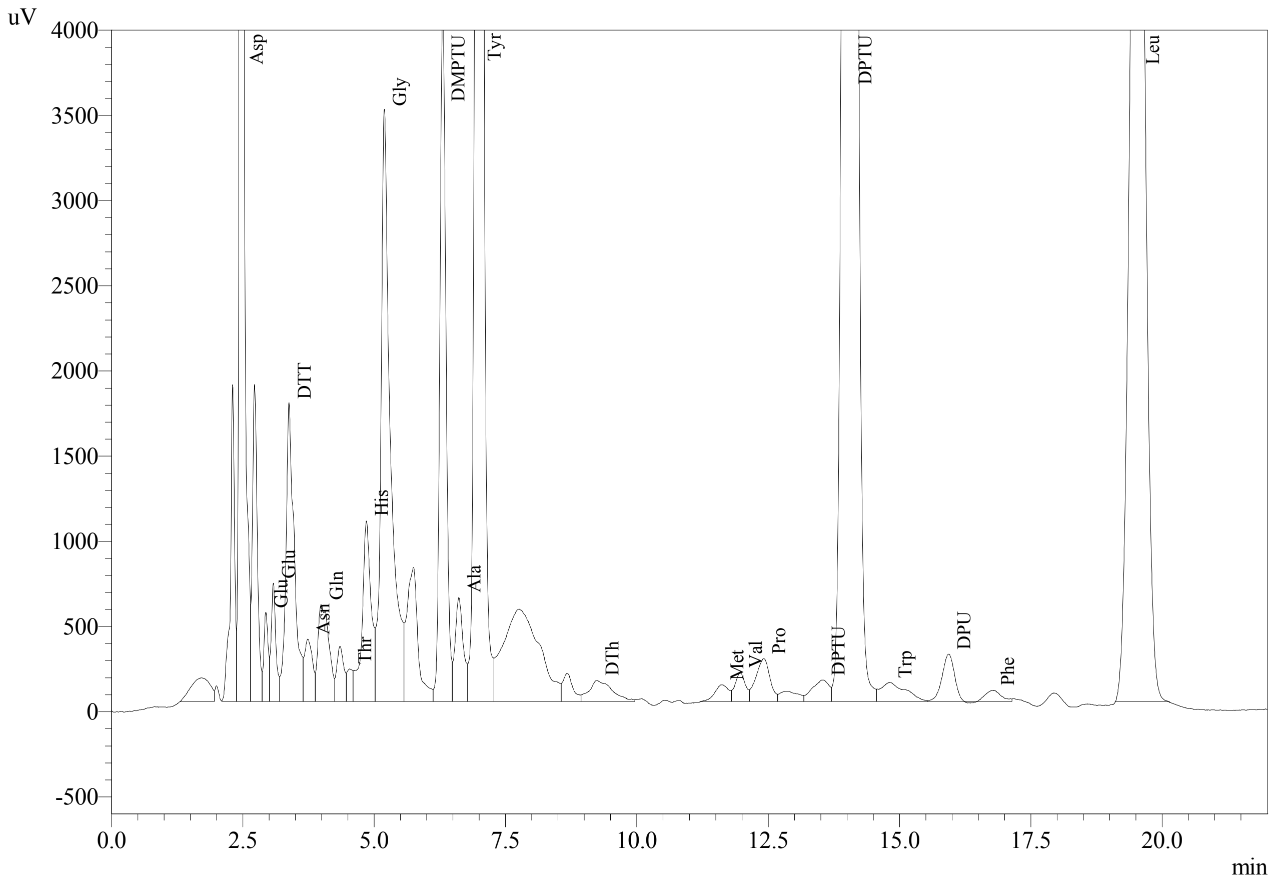

PeakTable
Detector A Ch1 269nm

Peak#	Name	Ret. Time	Area	Conc.
1		1.712	3584	
2		2.304	10424	
3	Asp	2.467	66005	128.410
4		2.722	12197	
5	Glu	2.936	3062	6.701
6	Glu	3.079	4412	9.656
7	DTT	3.377	19509	
8	Asn	3.735	3946	8.618
9	Gln	3.988	8373	17.684
10		4.347	3112	
11	Thr	4.533	1437	3.736
12	His	4.850	13000	28.933
13	Gly	5.192	44258	115.455
14		5.745	11861	
15	DMPTU	6.303	33452	
16	Ala	6.609	6780	16.239
17	Tyr	6.990	130569	311.365
18		7.753	26111	
19		8.672	2235	
20	DTh	9.233	3804	24.257
21	Met	11.614	1819	4.032
22	Val	11.965	2343	5.197
23	Pro	12.412	4737	13.126
24		12.852	1487	
25	DPTU	13.537	2821	
26	DPTU	14.049	301521	
27	Trp	14.812	3736	7.836
28	DPU	15.932	4591	

Peak#	Name	Ret. Time	Area	Conc.
29	Phe	16.760	1420	3.488
30	Leu	19.526	135037	299.229
Total			867640	

Data Filename : 5812A_070515+_02_D07.lcd
Sample Name : 5812-B
Method Filename : 5812A_070515+_02_SQ.LCM
Date Acquired : 07.05.2015 19:41:11
Background Filename :

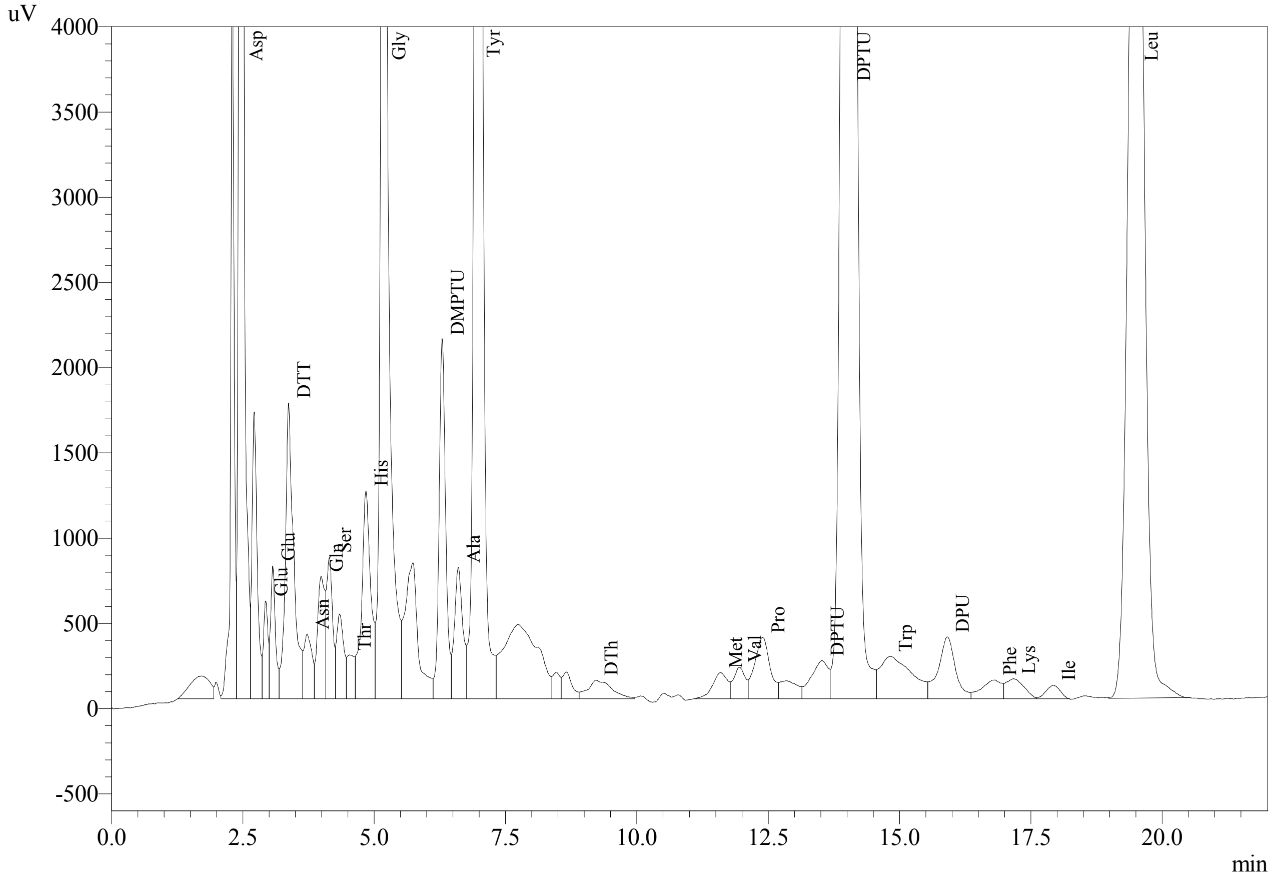

PeakTable
Detector A Ch1 269nm

Peak#	Name	Ret. Time	Area	Conc.
1		1.711	3555	
2		2.297	20000	
3	Asp	2.457	81659	158.863
4		2.714	11503	
5	Glu	2.933	3384	7.407
6	Glu	3.068	5102	11.167
7	DTT	3.369	19250	
8	Asn	3.721	4062	8.870
9	Gln	3.987	7183	15.170
10	Ser	4.144	6556	20.528
11		4.340	4752	
12	Thr	4.520	2559	6.657
13	His	4.841	14836	33.019
14	Gly	5.178	74719	194.920
15		5.733	14157	
16	DMPTU	6.292	18541	
17	Ala	6.599	8654	20.726
18	Tyr	6.977	112725	268.812
19		7.736	20126	
20		8.465	1523	
21		8.653	2009	
22	DTh	9.218	3429	21.870
23	Met	11.589	3012	6.678
24	Val	11.950	2872	6.371
25	Pro	12.391	7415	20.544
26		12.843	2406	
27	DPTU	13.521	5079	
28	DPTU	14.027	288045	

Peak#	Name	Ret. Time	Area	Conc.
29	Trp	14.817	10421	21.859
30	DPU	15.906	8232	
31	Phe	16.806	2873	7.060
32	Lys	17.167	2762	4.141
33	Ile	17.925	1445	3.143
34	Leu	19.498	134016	296.969
Total			908865	

Data Filename	: 5812A_070515+_02_D08.lcd
Sample Name	: 5812-B
Method Filename	: 5812A_070515+_02_SQ.LCM
Date Acquired	: 07.05.2015 20:29:36
Background Filename	:

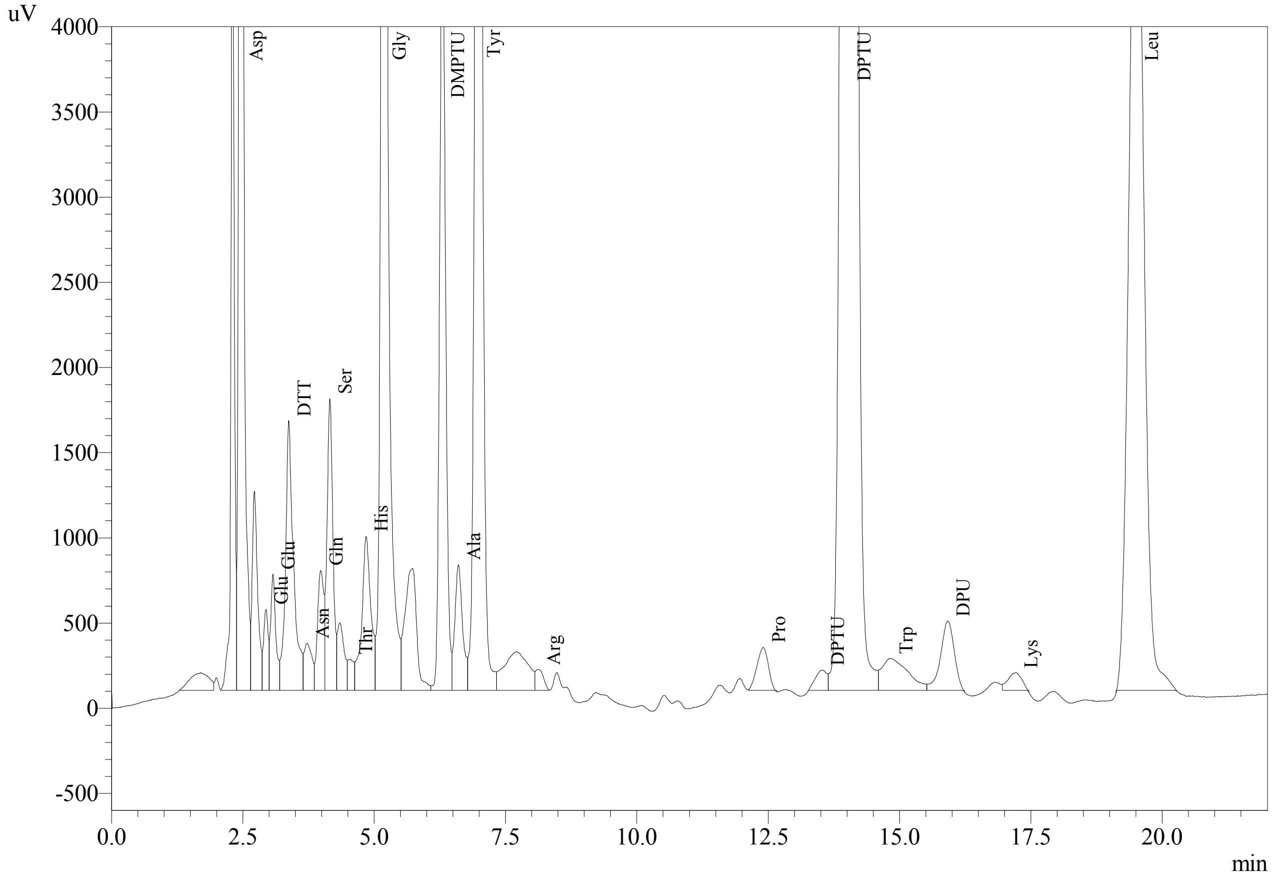

PeakTable
Detector A Ch1 269nm

Peak#	Name	Ret. Time	Area	Conc.
1		1.694	2642	
2		2.300	21565	
3	Asp	2.457	69936	136.056
4		2.718	8551	
5	Glu	2.938	2884	6.312
6	Glu	3.071	4733	10.359
7	DTT	3.372	16955	
8	Asn	3.720	2879	6.287
9	Gln	3.980	5994	12.659
10	Ser	4.154	13477	42.199
11		4.343	3559	
12	Thr	4.550	1469	3.820
13	His	4.845	10988	24.453
14	Gly	5.182	78821	205.621
15		5.726	11240	
16	DMPTU	6.297	36020	
17	Ala	6.601	7662	18.350
18	Tyr	6.981	82734	197.292
19		7.708	7301	
20	Arg	8.122	1267	3.062
21	Pro	12.403	3752	10.396
22	DPTU	13.528	1796	
23	DPTU	14.031	422302	
24	Trp	14.828	6450	13.529
25	DPU	15.915	7351	
26	Lys	17.210	1879	2.818
27	Leu	19.503	105452	233.672
Total			939657	

Data Filename : 5812A_070515+_02_D09.lcd
Sample Name : 5812-B
Method Filename : 5812A_070515+_02_SQ.LCM
Date Acquired : 07.05.2015 21:17:59
Background Filename :

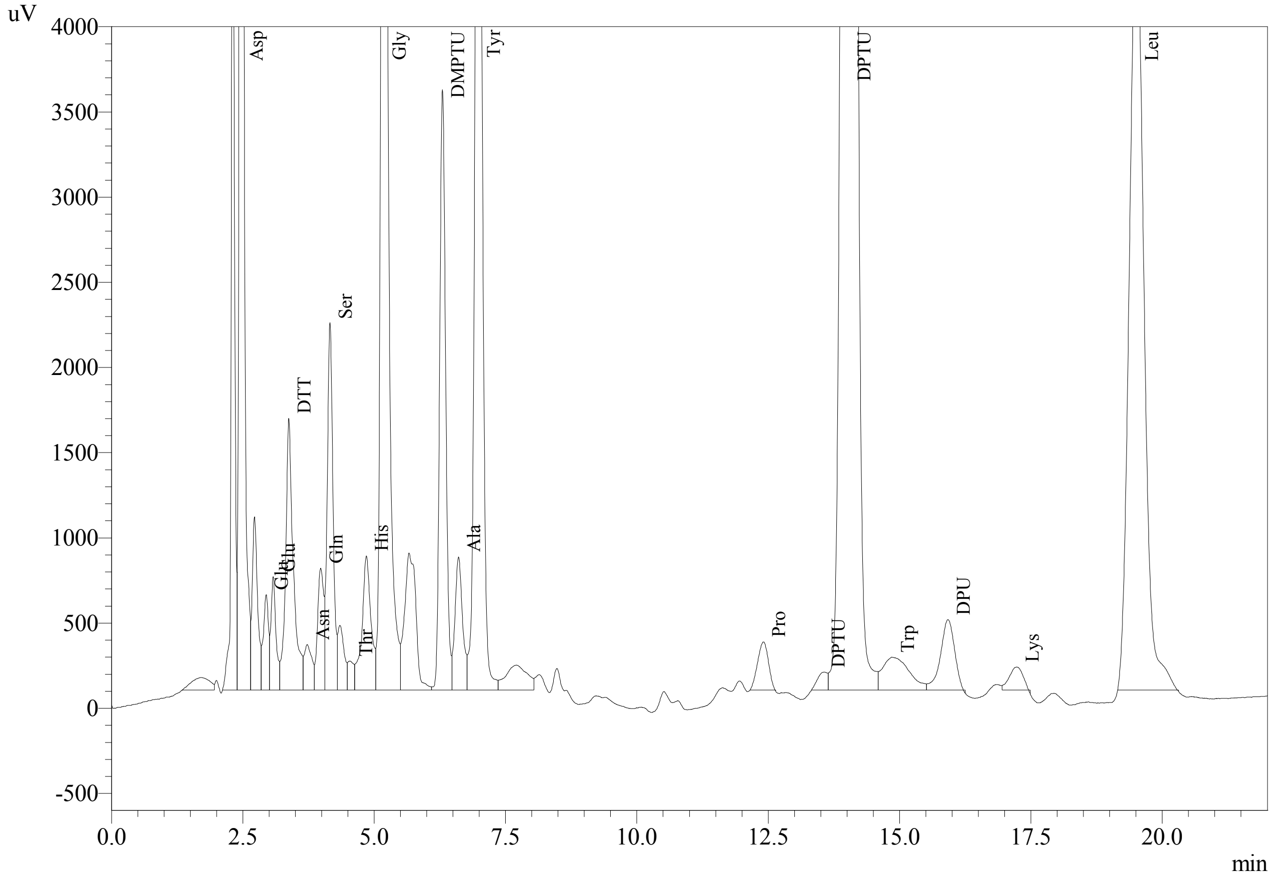

PeakTable
Detector A Ch1 269nm

Peak#	Name	Ret. Time	Area	Conc.
1		1.702	1819	
2		2.305	23763	
3	Asp	2.467	67095	130.529
4		2.720	7326	
5	Glu	2.943	3918	8.575
6	Glu	3.076	4500	9.849
7	DTT	3.374	16451	
8	Asn	3.724	2732	5.967
9	Gln	3.980	6006	12.684
10	Ser	4.156	16555	51.837
11		4.345	3209	
12	Thr	4.550	1365	3.551
13	His	4.849	9603	21.372
14	Gly	5.182	85044	221.853
15		5.661	12202	
16	DMPTU	6.298	28202	
17	Ala	6.603	7962	19.070
18	Tyr	6.982	66315	158.139
19		7.705	4214	
20	Pro	12.406	4053	11.229
21	DPTU	13.540	1389	
22	DPTU	14.032	355006	
23	Trp	14.861	6819	14.302
24	DPU	15.919	7732	
25	Lys	17.229	2458	3.685
26	Leu	19.505	88078	195.174
Total			833816	

Data Filename : 5812A_070515+_02_D10.lcd
Sample Name : 5812-B
Method Filename : 5812A_070515+_02_SQ.LCM
Date Acquired : 07.05.2015 22:06:23
Background Filename :

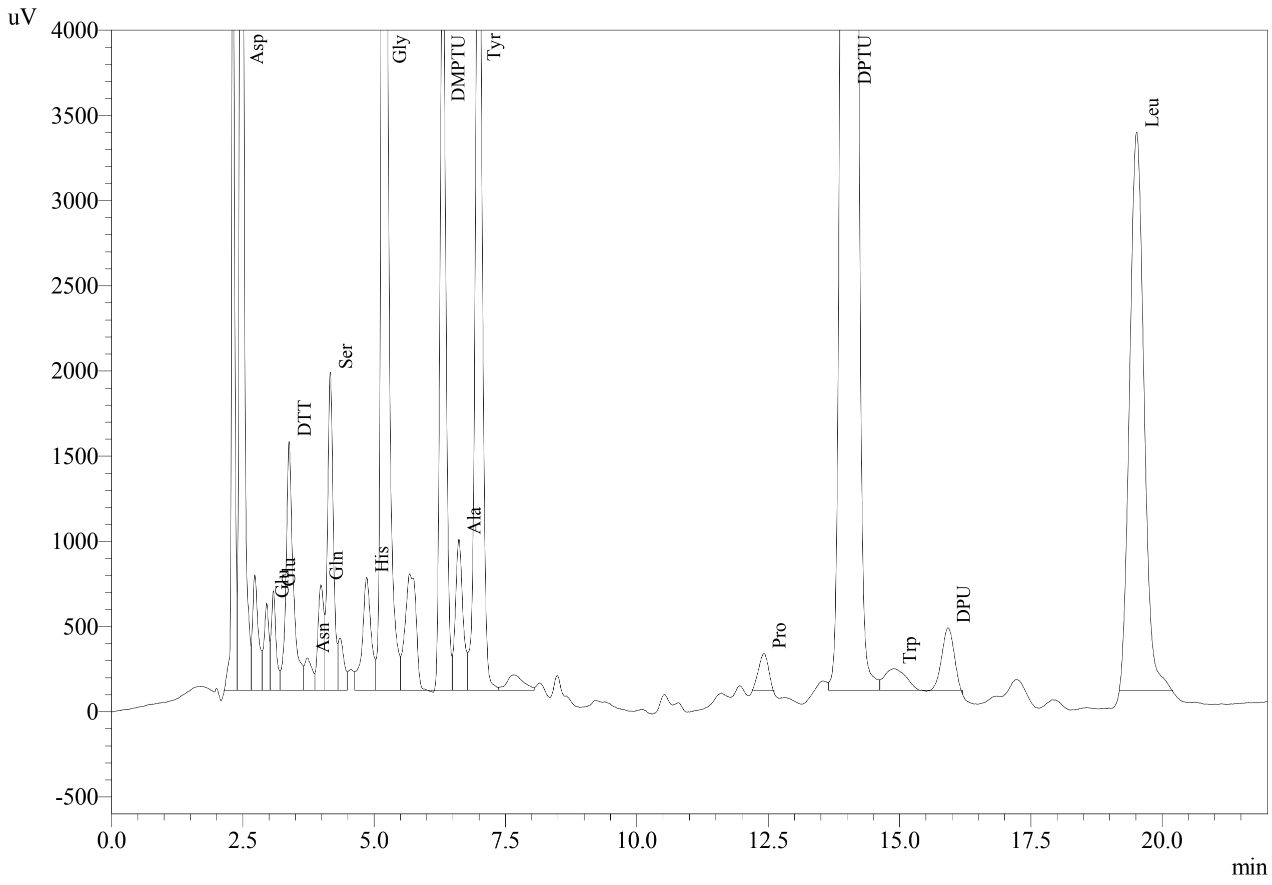

PeakTable
Detector A Ch1 269nm

Peak#	Name	Ret. Time	Area	Conc.
1		2.309	20150	
2	Asp	2.469	52461	102.060
3		2.726	5275	
4	Glu	2.953	3336	7.301
5	Glu	3.081	3747	8.200
6	DTT	3.379	14060	
7	Asn	3.726	1891	4.130
8	Gln	3.984	4839	10.219
9	Ser	4.162	14423	45.163
10		4.347	2356	
11	His	4.854	7767	17.286
12	Gly	5.188	74488	194.317
13		5.674	10041	
14	DMPTU	6.304	34887	
15	Ala	6.610	8820	21.124
16	Tyr	6.987	47192	112.537
17		7.667	2016	
18	Pro	12.418	2782	7.707
19	DPTU	14.036	377407	
20	Trp	14.895	3624	7.601
21	DPU	15.919	5939	
22	Leu	19.510	62402	138.277
Total			759903	

Data Filename	: 5812A_070515+_02_D11.lcd
Sample Name	: 5812-B
Method Filename	: 5812A_070515+_02_SQ.LCM
Date Acquired	: 07.05.2015 22:54:47
Background Filename	:

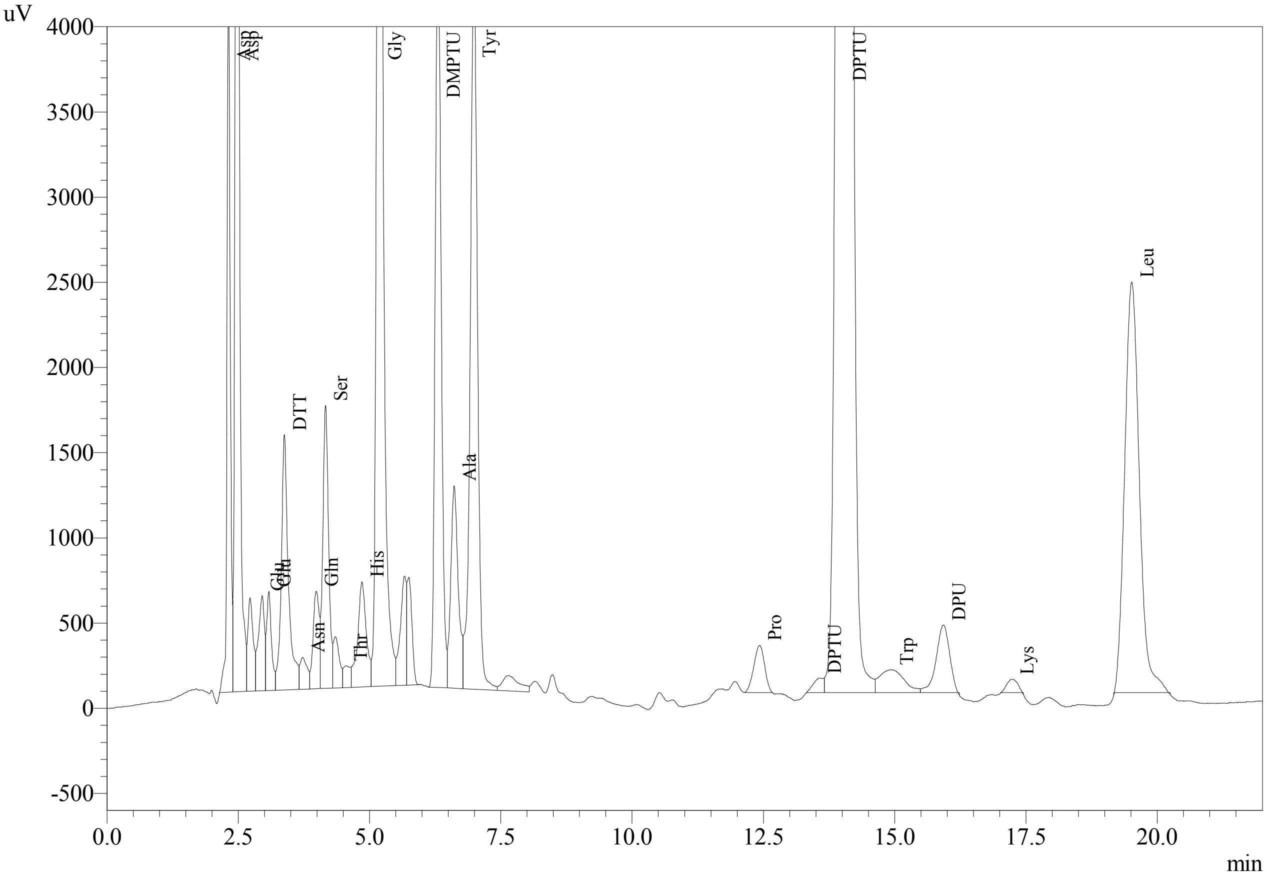

PeakTable
Detector A Ch1 269nm

Peak#	Name	Ret. Time	Area	Conc.
1	Asp	2.311	18279	35.561
2	Asp	2.473	46568	90.595
3		2.724	3932	
4	Glu	2.953	4525	9.903
5	Glu	3.081	3801	8.319
6	DTT	3.376	14122	
7	Asn	3.725	1801	3.934
8	Gln	3.983	4667	9.857
9	Ser	4.160	12821	40.147
10		4.349	2477	
11	Thr	4.540	1169	3.041
12	His	4.853	7158	15.930
13	Gly	5.187	63670	166.095
14		5.664	5288	
15		5.745	3983	
16	DMPTU	6.302	35048	
17	Ala	6.609	11890	28.476
18	Tyr	6.984	40557	96.716
19		7.628	2008	
20	Pro	12.425	3950	10.945
21	DPTU	13.560	1168	
22	DPTU	14.036	374386	
23	Trp	14.931	4362	9.150
24	DPU	15.924	7058	
25	Lys	17.226	1122	1.683
26	Leu	19.510	46880	103.883
Total			722692	

Data Filename : 5812A_070515+_02_D12.lcd
Sample Name : 5812-B
Method Filename : 5812A_070515+_02_SQ.LCM
Date Acquired : 07.05.2015 23:43:12
Background Filename :

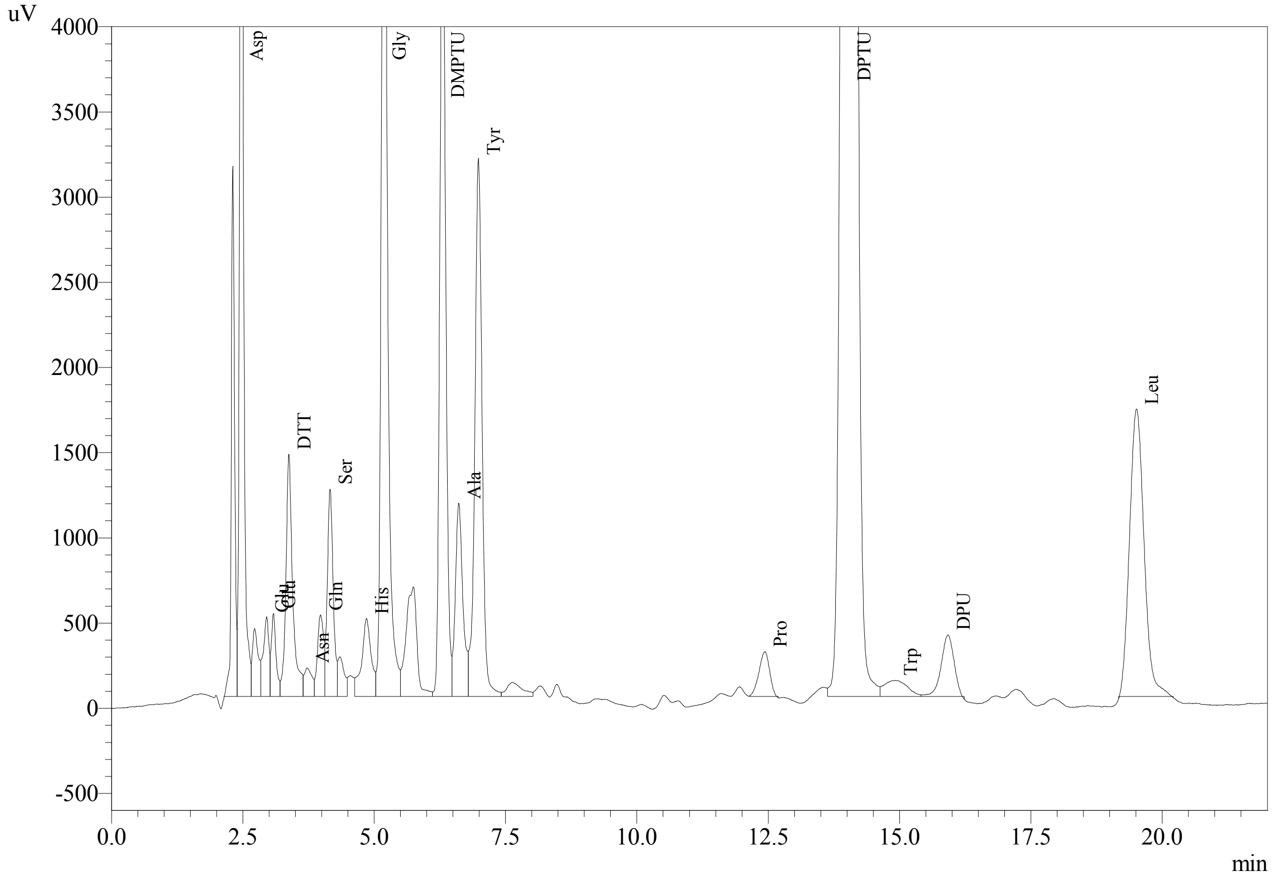

PeakTable
Detector A Ch1 269nm

Peak#	Name	Ret. Time	Area	Conc.
1		2.306	13594	
2	Asp	2.467	32856	63.920
3		2.722	3145	
4	Glu	2.951	3540	7.748
5	Glu	3.080	3122	6.833
6	DTT	3.374	12860	
7	Asn	3.723	1729	3.776
8	Gln	3.976	3949	8.340
9	Ser	4.158	9557	29.925
10		4.346	2002	
11	His	4.851	5803	12.915
12	Gly	5.184	48476	126.459
13		5.744	9449	
14	DMPTU	6.300	37589	
15	Ala	6.607	11441	27.401
16	Tyr	6.982	30420	72.543
17		7.628	1742	
18	Pro	12.435	3699	10.248
19	DPTU	14.034	350132	
20	Trp	14.917	2893	6.069
21	DPU	15.918	6154	
22	Leu	19.507	32494	72.005
Total			626648	
